# A comprehensive characterization of the nuclear microRNA repertoire of post-mitotic neurons

**DOI:** 10.3389/fnmol.2013.00043

**Published:** 2013-11-26

**Authors:** Sharof A. Khudayberdiev, Federico Zampa, Marek Rajman, Gerhard Schratt

**Affiliations:** Biochemisch-Pharmakologisches Centrum, Institut für Physiologische Chemie, Philipps-Universität MarburgMarburg, Germany

**Keywords:** miRNA, isomiR, neuronal development, plasticity, deep sequencing, microarray

## Abstract

MicroRNAs (miRNAs) are small non-coding RNAs with important functions in the development and plasticity of post-mitotic neurons. In addition to the well-described cytoplasmic function of miRNAs in post-transcriptional gene regulation, recent studies suggested that miRNAs could also be involved in transcriptional and post-transcriptional regulatory processes in the nuclei of proliferating cells. However, whether miRNAs localize to and function within the nucleus of post-mitotic neurons is unknown. Using a combination of microarray hybridization and small RNA deep sequencing, we identified a specific subset of miRNAs which are enriched in the nuclei of neurons. Nuclear enrichment of specific candidate miRNAs (miR-25 and miR-92a) could be independently validated by Northern blot, quantitative real-time PCR (qRT-PCR) and fluorescence *in situ* hybridization (FISH). By cross-comparison to published reports, we found that nuclear accumulation of miRNAs might be linked to a down-regulation of miRNA expression during *in vitro* development of cortical neurons. Importantly, by generating a comprehensive isomiR profile of the nuclear and cytoplasmic compartments, we found a significant overrepresentation of guanine nucleotides (nt) at the 3′-terminus of nuclear-enriched isomiRs, suggesting the presence of neuron-specific mechanisms involved in miRNA nuclear localization. In conclusion, our results provide a starting point for future studies addressing the nuclear function of specific miRNAs and the detailed mechanisms underlying subcellular localization of miRNAs in neurons and possibly other polarized cell types.

## Introduction

MicroRNAs (miRNAs) are an important class of small regulatory non-coding RNAs with a size of 18–25 nucleotides (nt). The canonical miRNA biogenesis pathway starts with the generation of the primary miRNA (pri-miRNA) transcript by RNA polymerase II mediated transcription. The pri-miRNA transcript is cleaved by the microprocessor complex, containing among other proteins Drosha and Di George Syndrome critical region gene 8 (DGCR8) proteins, which results in ~70 nt hairpin-like precursor miRNAs (pre-miRNA). Pre-miRNAs are subsequently exported to the cytoplasm by the nuclear export receptor Exportin-5 (Zeng and Cullen, 2004), where they are further cleaved by Dicer to produce an intermediate RNA duplex. One strand of this duplex (known as guide miRNA) binds to an Argonaute family protein (AGO) 1–4, the core component of the miRNA-associated RNA-induced silencing complex (miRISC). MiRISC mainly functions in the cytoplasmic compartment by translational inhibition and/or degradation of target mRNAs. MiRNAs are implicated in many steps of neuronal development and the function of mature neurons, including synaptic plasticity, learning and memory (Fiore et al., 2011). Interestingly, several recent studies suggest that miRNAs, in addition to their well-defined role in the cytoplasm, may also be involved in the regulation of gene expression in the nucleus of mammalian cells.

First, it was shown that miRNAs are present in the nuclear compartment. Some of them are even enriched in the nuclei or nucleoli of cancer cell lines (Hwang et al., 2007; Liao et al., 2010; Park et al., 2010; Li et al., 2013), myoblasts (Politz et al., 2009) and neural stem cells (Jeffries et al., 2011). Second, the key components of the miRNA pathway, such as Ago (Tan et al., 2009), Dicer (Sinkkonen et al., 2010) and multiple glycine/tryptophan repeat containing protein - GW182 (Till et al., 2007; Nishi et al., 2013), are detected in the nucleus. Third, Ago proteins associate with splicing factors (Ameyar-Zazoua et al., 2012) and regulate siRNA-mediated alternative splicing (Allo et al., 2009). Fourth, some miRNAs were shown to post-transcriptionally regulate gene expression in the nucleus (Hansen et al., 2011; Tang et al., 2012). Finally, several miRNAs (and siRNAs) were identified to control gene expression by binding to the promoter of target genes, thereby triggering epigenetic changes, such as DNA methylation (Morris et al., 2004) and histone modification (Kim et al., 2008; Place et al., 2008; Benhamed et al., 2012).

Epigenetic modifications and alternative mRNA splicing, apart from being important in neuronal differentiation, are also implicated in activity-dependent gene expression in mature neurons (Norris and Calarco, 2012; Zovkic et al., 2013), an essential mechanism for synaptic plasticity, learning and memory. Furthermore, genes undergoing alternative mRNA splicing are overrepresented in the brain (Yeo et al., 2004), suggesting that specific molecular mechanisms that lead to transcript diversity must be present in the brain. However, whether miRNAs can regulate gene expression by any of the aforementioned mechanisms in the neuronal nucleus is not known. A prerequisite for the study of miRNA function in the nucleus of post-mitotic neurons is the *a priori* knowledge of the nuclear miRNA repository. However, to date nuclear miRNAs have only been identified from proliferating cells, and it can be expected that terminally differentiated cells like neurons have a completely different miRNA expression profile.

In the present study, using microarray and deep sequencing technologies, we identified miRNAs which are enriched in the nuclei of rat primary cortical neurons. Our results suggest that employing a combination of microarray and deep sequencing technologies to determine nuclear-enriched miRNAs can yield more accurate results than using each method separately. Accordingly, we could validate differential expression of specific nuclear-enriched miRNAs by Northern blot, quantitative real-time PCR (qRT-PCR) and fluorescence *in situ* hybridization (FISH). By cross-comparison to published reports we observed that expression levels of nuclear-enriched miRNAs in general decline during development of neurons, suggesting that these miRNAs could play a role in early developmental stages of neurons. Importantly, by generating a comprehensive isomiR profile of the nuclear and cytoplasmic compartments, we found that the most 3′-terminal nucleotide of miRNA species is a robust predictor of nuclear enrichment. In conclusion, our results provide a roadmap for future studies addressing the detailed mechanisms underlying subcellular localization of miRNAs in neurons and possibly other polarized cell types.

## Materials and methods

### Primary neuronal culture

Primary cortical and hippocampal neuron cultures were prepared from embryonic Day 18 (E18) Sprague-Dawley rats (Charles River Laboratories) as previously described (Schratt et al., 2006). Cortical and hippocampal cultures were maintained in Neurobasal (NB) medium containing 2% B27 supplement, penicillin-streptomycin (100 U/ml penicillin, 100 μg/ml streptomycin), and GlutaMax (1 mM). All reagents were purchased from Life Technologies. Glia-depleted cultures were obtained by supplementing FUDR solution (10 μM) starting from day *in vitro* 0 (DIV0). FUDR solution was prepared by mixing equimolar amount of fluorodeoxyuridine (Sigma) and uridine (Sigma). Glia-enriched cultures were maintained in the standard medium, except B27 supplement was exchanged to 10% FBS (Life Technologies). When indicated, cells were treated for 2 h with 40 ng/mL of BDNF (PeproTech) or 55 mM of KCl solution.

### Nuclear fractionation protocol

For nuclear fractionation, 40 million cells from cortical cultures at DIV7 were used. Cells were washed once with 10 mL of ice-cold 1 × Phosphate buffered saline (PBS; Life Technologies) and were scraped into ice-cold 1 × PBS using cell lifters (Corning). Then cells were pelleted by centrifugation at 100 g speed for 5 min at 4°C. Subsequently, cell pellet was resuspended in 600 μl of ice-cold hypotonic homogenization buffer [HHB; 10 mM KCl, 1.5 mM MgCl_2_, 1 mM Na-EDTA, 1 mM Na-EGTA, 10 mM Tris-HCl pH = 7.4, 1 mM DTT, 2 u/μl RNasin Plus RNase inhibitor (Promega)] and was incubated on ice for 30 min. After supplying cell suspension with 600 μl of 0.2% Igepal CA630 containing HHB, it was homogenized with 40 stokes in a Dounce potter. From the obtained cell lysate, nuclear and cytoplasmic fractions were separated by centrifugation at 720 g speed for 5 min at 4°C. The nuclear fraction (pellet) was washed three times with 1.5 mL of isotonic homogenization buffer (IHB; HHB, supplemented with 250 mM sucrose). The total RNA from nuclear (pellet) and cytoplasmic (supernatant) fractions was extracted using peqGOLD TriFast reagent (Peqlab) per manufacturer's instructions. On average, 15–20% of the total RNA derived from the fractionation originated from the nucleus. For determination of nuclear and cytoplasmic protein markers, the nuclear pellet obtained after washes with IHB was resuspended in RIPA buffer [10 mM NaCl, 1% Triton X-100, 0.5% Sodiumdeoxycholate, 1 mM EGTA, 0.05% SDS, 50 mM Tris-HCl pH = 8.0, fresh 5x protease inhibitor cocktail (Roche)].

### Western blotting

Western blotting was performed as previously described (Siegel et al., 2009). The following primary antibodies were used: anti-HDAC2-rabbit monoclonal (Abcam) and anti-beta Actin-mouse monoclonal (Sigma).

### RNA extraction, size selection of small RNAs and microarray procedure

Twelve microgram of total RNA from nuclear and cytoplasmic fractions was supplemented with spike-in oligoribonucleotides (18 nt, 5-Phos-AGCGUGUAGGGAUCCAAA-3; 24 nt, 5-Phos-GGCCAACGUUCUCAACAAUAGUGA-3; 30 nt, 5-Phos-GGCAUUAACGCGGCCGCUCUACAAUAGUGA-3; 50 femtomoles of each; http://bartellab.wi.mit.edu/protocols.html) and mixed with the same volume of Gel loading buffer II (Life Technologies). RNA was separated using denaturing urea 15% PAGE gel (SequaGel System, National Diagnostics), which was run in 1 × TBE (89 mM Tris/89 mM Borate/2 mM EDTA) buffer at 30 Watts. Gel was stained with 2 × SYBR GOLD dye (Life Technologies; in 1 × TBE) for 10 min and gel pieces corresponding to small RNAs of 15–35 nt size were cut out. Small RNAs were eluted by incubation of gel pieces in 300 mM NaCl solution overnight at 4°C with constant rotation. Precipitation of RNA was carried out by addition of 2.5–3 volume of 100% EtOH to a supernatant and incubation at −20°C for at least 2 h. Pellet was resuspended in 20 μl of DEPC-treated H_2_O. For miRNA profiling analysis, 14 μl of small RNA, obtained from each sample, were sent to microRNA Microarray Service provided by LC Sciences (Texas, USA). In brief, three biological replicates of nuclear fractionated samples (three nuclear and three cytoplasmic samples) were labeled with Cy3 (nuclear) and Cy5 (cytoplasmic), and then were hybridized on a single microarray chip (dual-sample hybridization). The signal values were derived by background subtraction and global normalization. A transcript to be listed as detectable should have met at least two conditions: signal intensity higher than 3 × (background standard deviation) and spot CV < 0.5. CV was calculated by (standard deviation)/(signal intensity). When repeating probes were present on an array, a transcript was listed as detectable only if the signals from at least 50% of the repeating probes were above detection level. The data obtained from LC Sciences was further normalized to a signal intensity value of 24 nt spike-in oligoribonucleotides. The probes on the array were based on miRBase version 16 that contained 679 rat miRNAs. For expression analysis, only miRNAs that possessed average signal intensity values of at least 35 (higher than log_2_[average signal intensity] = 5) after background subtraction (where signal intensity values of miRNAs that were same as the background signal were considered as zero), in either of the cellular fractions, were considered. Nuclear enrichment score (NEnS) was calculated by taking logarithm base 2 of the ratio of (average nuclear signal intensity value)/(average cytoplasmic signal intensity value). Statistical analysis was performed on signal intensity values with Student's *t*-test (two-tail, paired). The calculation of Pearson's coefficient between different microarray datasets was performed in Excel (Analysis ToolPak add-in) and was based on log_2_ transformed signal intensity values of miRNAs.

### Deep sequencing

Small RNA libraries were constructed and sequenced by EMBL genomic core facility (Heidelberg, Germany). In brief, four small RNA libraries (2 nuclear and 2 cytoplasmic) representing two biological replicates were prepared using small RNA sample prep assay (Illumina) as per manufacturer's instructions. Each of the small RNA libraries was sequenced for 36 cycles in a single lane of one Illumina HiSeq flow cell. Raw sequencing reads were trimmed from 3′ adapter (TCGTATGCCGTCTTCTGCTTG) and filtered according to quality using default parameters of Fastx-Toolkit for fastq data on a Galaxy, a web-based genome analysis tool [(Goecks et al., 2010); https://main.g2.bx.psu.edu/]. Sequencing reads that contained only adapter sequence or those that initially (before trimming) did not contain adapter sequence, as well as reads shorter than 15 nt were discarded. Furthermore, only reads that have at least two identical sequence counts in each of the libraries were considered for analysis (“clean reads”). Clean reads were mapped to the rat mature miRNAs (miRBase v19) using default parameters (one mismatch, 3 nt in the 3′ or 5′-trimming variants, 3 nt in the 3′-addition variants) of Miraligner software (Pantano et al., 2010). The rest of the unmapped reads were first mapped to rat premiRNAs (miRBase v19) and then to other classes of non-coding RNAs [snoRNAs, snRNAs, rRNAs, tRNAs, mitochondrial tRNAs, mitochondrial rRNAs, miscRNAs; sequences were retrieved from Ensembl genome database (rn4) using BioMart portal, http://central.biomart.org/], piRNAs (http://www.ncrna.org/frnadb/, http://www.noncode.org), mRNAs (mRNA_coding sequence, 3′UTR, -1000_transcription_start_site+5UTR; sequences were retrieved from Ensembl genome database (rn4) using BioMart portal, http://central.biomart.org/] and finally to rat genome (ftp://ftp.ccb.jhu.edu/pub/data/bowtie_indexes/; USCS rn4) in a sequential order using bowtie-0.12.8 software (Langmead, 2010) allowing up to 2 mismatches. All read counts that were mapped to the sequences from aforementioned RNA/DNA databases were used to normalize between nuclear and cytoplasmic small RNA libraries. After normalization, miRNAs represented by at least 100 reads in one of the cellular compartments were considered for further analysis. Nuclear enrichment score (NEnS) was calculated by taking logarithm base 2 of the ratio of (average nuclear read count)/(average cytoplasmic read count). The rank based comparison of microarray and deep sequencing was performed by Rank Sum function of RankProdIt [http://strep-microarray.sbs.surrey.ac.uk/RankProducts/; (Laing and Smith, 2010)].

### Quantitative real-time PCR

The total RNA extraction from neuronal cultures was performed using peqGOLD TriFast reagent per manufacturer's instructions. RNA samples were treated with TURBO DNase (Ambion). For detection of small nuclear RNAs (U1, U4, U6) and mRNAs (GAPDH), 200 ng of total RNA sample was reverse transcribed with iScript cDNA synthesis kit (Bio-Rad) and quantitative real-time PCR (qRT-PCR) was performed on the StepOnePlus Real-Time PCR System (Applied Biosystems), using iTaq SYBR Green Supermix with ROX (Bio-Rad). For detection of mature miRNAs, 50 ng of total RNA sample was reverse transcribed using the TaqMan MicroRNA Reverse Transcription Kit and qRT-PCR was performed on the StepOnePlus Real-Time PCR System (Applied Biosystems), using TaqMan MicroRNA Assay (Applied Biosystems). Each sample was measured in dub—or triplicates. qRT-PCR data from nuclear fractionated samples were analyzed by 2^−dCt^ [2^−(NUC Ct−CYT Ct)^] method (ΔCt method). Data obtained from whole-cell RNA (developmental, neuron, glia-specific expression) were analyzed by ΔΔCt method, where Ct values were first normalized to an internal control (e.g., U6) and then to the reference sample, which was arbitrarily set to 1. For statistical analysis (Student's and Welch's *t*-tests) the data, which was normalized only to U6 was used. Primers used for the qRT-PCR are provided as supplementary data (Table S8).

### Northern blot

From ten to twenty microgram of total RNA were separated using denaturing urea 15% PAGE gel (Mini-PROTEAN system; Bio-Rad) in 1x TBE and blotted onto a GeneScreen Plus nylon membrane (PerkinElmer) in pre-cooled 0.5x TBE. Radioactively labeled Decade marker (Ambion) was used as molecular marker. RNAs were crosslinked to the membrane by UV irradiation (1200 mJ), followed by baking of the membrane for 30 min at 80°C. The membrane was pre-incubated in hybridization buffer (5 × SSC, 20 mM Na_2_HPO_4_ (pH = 7.2), 7% SDS, 2 × Denhardt's solution, 40 μg/mL salmon sperm DNA) for at least 2 h at 50°C at constant rotation, followed by incubation overnight at 50°C in hybridization buffer containing the denatured [32P] labeled DNA probe. The membrane was washed twice for 10 min and twice for 30 min at 50°C with non-stringent wash solution (3 × SSC, 25 mM NaH_2_PO_4_ (pH = 7.5), 5% SDS, 10 × Denhardt's solution) and once for 5 min at 50°C with stringent wash solution (1 × SSC, 1% SDS). Signals were detected by autoradiography using the Cyclone Plus Phosphor Imager (PerkinElmer). The membrane was stripped (0.1% SDS, 5 mM Na-EDTA, preheated to 95°C) for 1 h and re-used several times to detect additional miRNAs and U6 snRNA. DNA probes are provided as supplementary data (Table S8).

### Fluorescence *in-situ* hybridization (FISH)

FISH was performed on dissociated hippocampal neurons at DIV5. Cells were fixed with 4% PFA/4% sucrose/DEPC-PBS for 15 min at room temperature and washed three times with DEPC-PBS. After permeabilization using 0.2% Tween/DEPC-PBS for 2 min, cells were washed twice with DEPC-PBS and treated for 5 min with 0.1 M TEA (Triethanolamine-acetic acid in DEPC-H_2_O, pH 8.0) and for 10 min with freshly prepared 0.25% Acetic Anhydride in 0.1 M TEA. Cells were washed three times with DEPC-PBS and pre-incubated in hybridization buffer at 55°C for 1 h. Subsequently, hybridization was carried out overnight at 55°C, using hybridization buffer supplemented with denatured (5 min 85°C, 5 min on ice) DIG (or FITC)-labeled LNA probes (Exiqon; 5 pmol per well in the 24-well format) directed against relevant miRNA. Cells were washed twice in 2x SSC and twice in 0.2x SSC, 30 min each. After two washes with PBS, cells were permeabilized with 0.2% Tween/PBS for 2 min and washed again twice with PBS. Depending on the condition, for signal amplification and co-immunostaining, cells were incubated with first set of antibody dilutions [anti-MAP2–mouse (Sigma) + anti-DIG–FITC (Roche) for U6, miR-25 and miR-92a; anti-MAP2–mouse + anti-FITC–Alexa488–rabbit (Life Technologies) for miR-9] in blocking solution [0.5% Blocking Reagent in PBS (Roche)] for 1.5 h at room temperature. After four washes with PBS, second set of antibodies (anti-Mouse–Alexa546 (Life Technologies) + anti-FITC–Alexa488–rabbit; anti-Mouse–Alexa546, respectively) was applied for 30 min at room temperature. Then cells were washed four more times with PBS and incubated in the last antibody [anti-Rabbit-Alexa488 (Life Technologies)] solution for 30 min. Cells were washed three times with PBS (second wash with Hoechst dye—1:20,000) and mounted on microscope slides using Aqua- Poly/Mount (Polysciences). FISH experiments were analyzed using the 63x objective of the LSM 5 Pascal laser scanning confocal microscope (Zeiss), with identical settings for specific probes. For z-stacks, three consecutive optical sections were taken at a 0.4 μm interval with a resolution of 1024 × 1024 pixels. Maximum projections of the z-stack images were used for subsequent analysis of the signal intensities in nucleus and cytoplasm with the ImageJ software. LNA probes are provided as supplementary data (Table S8).

### Immunocytochemistry

Immunostaining of endogenous MAP2 anti-MAP2–mouse (Sigma) and GFAP [anti-GFAP-rabbit (DakoCytomation)] in dissociated hippocampal neurons (DIV18) was performed as described (Siegel et al., 2009).

### Developmental expression score

DES was calculated by log2 transforming the ratio of miRNA expression values obtained from prefrontal cortex of post-natal Day 3 (P3) and embryonic Day 10 (E10) rats in the published report by Yao and colleagues (Yao et al., 2012).

### IsomiR analysis

IsomiRs with at least 10 reads in one of the cellular fractions were considered for analysis presented in Figures 7B,C. The relative nuclear enrichment score (rNEnS) was calculated as a ratio of nuclear vs. cytoplasmic percentage proportion of a certain miRNA variants (isomiRs) and therefore should be distinguished from NES (which is an absolute value). For example, miRNA isoforms of miR-1 are isomiR-1.1 (constitutes 20% of miR-1 with 20 read counts in the nucleus; 30% with 60 reads in the cytoplasm), isomiR-1.2 (30% and 30 reads, nucleus; 50% and 100 reads, cytoplasm) and isomiR-1.3 (50% and 50 reads, nucleus; 20% and 40 reads, cytoplasm). The rNEnSs for these isomiRs are 20/30 = 0.66, 30/50 = 0.6 and 50/20 = 2.5, respectively, although NEnS for the same isomiRs constitute 20/60 = 0.33, 30/100 = 0.3, and 50/40 = 1.25, respectively. The usage of rNEnS allows to determine the impact of 3′-terminal nucleotide modification of isomiRs on preferential nuclear localization, since it calculates overall proportion of isomiR read counts in the specific cellular compartment independent of whether it is underrepresented in the other cellular compartment. The frequency of nt at 3′ last 5 nt was calculated using WebLogo [(Crooks et al., 2004); http://weblogo.berkeley.edu/].

### Statistical analysis

Experiments are reported as mean ± standard deviation (*SD*) and based on three (if not otherwise stated) independent replications. Statistical significance was calculated using Student's (for samples with equal variance) and Welch's *t*-tests (for samples with unequal variance), and for multiple comparisons Bonferroni correction was applied (Benjamini et al., 2001).

## Results

### Microarray profiling of nuclear and cytoplasmic miRNAs

**Figure 1:**
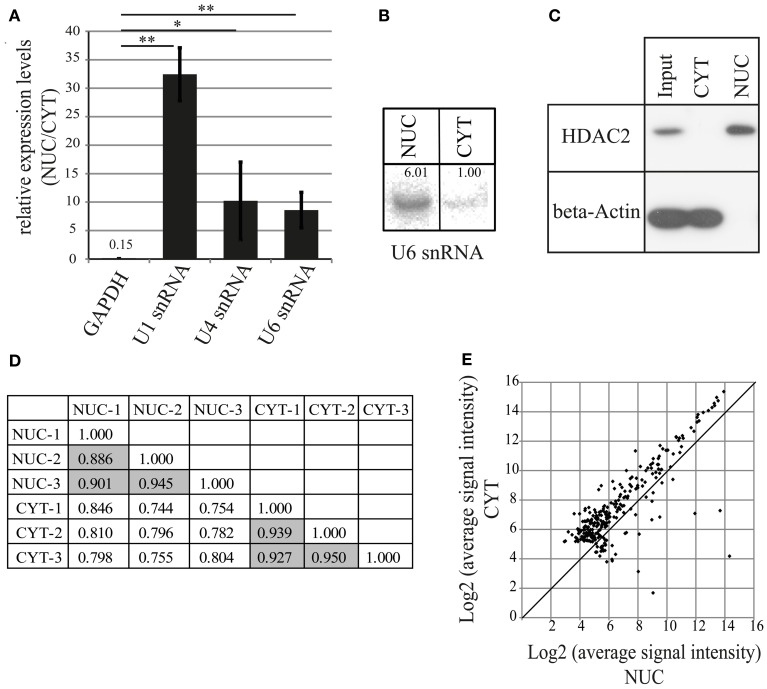
**MiRNA profiling by microarray after nucleo-cytoplasmic fractionation of neurons. (A)** qRT-PCR analysis of marker genes to validate the fractionation protocol. The fold enrichment (y-axis) of marker genes in the nucleus was calculated by the 2^−dCt^ [2^−(NUC Ct−CYT Ct)^] method. Bar plots show mean ± standard deviation (*SD*; *n* = 3). Statistical significance was determined using Student's *t*-test with Bonferroni correction (^*^p < 0.05; ^**^p < 0.01). **(B)** Northern blot analysis of the nuclear marker U6 snRNA in nuclear and cytoplasmic fractions. Intensity of the signal was quantified using ImageJ. **(C)** Detection of nuclear (HDAC2, histone deacetylase 2) and cytoplasmic (beta-Actin) marker proteins in the subcellular fractions using Western blotting assay. Whole cell lysate was used as an input sample. **(D)** Comparison of different biological replicates from microarray experiments. Pearson's correlation coefficients between indicated samples are shown. Data on gray background represents correlation coefficients for biological replicates from the same cellular fraction. **(E)** Distribution of miRNA expression in the nucleus and the cytoplasm. Scatterplot of log_2_ transformed signal intensity values for miRNAs from nuclear (x-axis) and cytoplasmic (y-axis) fractions (267). Dots above the diagonal indicate cytoplasmic enrichment, below, nuclear enrichment of the respective miRNAs.

To characterize miRNAs preferentially localizing to neuronal nuclei, we decided to undertake a biochemical fractionation approach that separates the nuclear and cytoplasmic compartments of rat primary cortical neurons cultured for 7 days *in vitro* (DIV). After isolation of total RNA from both compartments, the efficacy of nuclear fractionation was determined by the quantification of expression levels of small nuclear RNAs (snRNA U1, U4, U6; all strictly localized in the nucleus) and Glyceraldehyde 3-phosphate dehydrogenase (GAPDH) mRNA by qRT-PCR (Figures 1A, S1A). As expected, snRNAs were highly enriched in the nuclear compartment, whereas GAPDH mRNA was strongly depleted. Similar to the results obtained from qRT-PCR, we observed a 6-fold enrichment in the nuclear compartment for U6 snRNA with Northern blot assay (Figure 1B). Furthermore, the results from Western blotting showed exclusive expression of the protein markers HDAC2 and beta-Actin in the nuclear and cytoplasmic fraction, respectively (Figure 1C). Together, these results demonstrate that the used fractionation protocol can effectively separate nuclear and cytoplasmic compartments.

As a common practice, the raw data obtained from high throughput methods such as microarray are first normalized before the differential expression between two samples is calculated. Since we wanted to calculate the absolute enrichment of miRNAs in the nuclear compartment compared to the cytoplasmic compartment, we supplemented total RNA samples with spike-in oligoribonucleotides (18 nt, 24 nt, 30 nt) for normalization. Furthermore, in order to detect hybridization signals originating primarily from mature miRNAs, we size-selected total small RNAs (from 15 to 35 nt) from equal amounts (12 μg) of nuclear and cytoplasmic total RNA by 15% denaturing urea polyacrylamide gel electrophoresis (PAGE).

To determine expression levels of nuclear and cytoplasmic mature miRNAs, size-selected small RNA samples (3 nuclear and 3 cytoplasmic samples) were analyzed by miRNA microarrays (LCSciences), containing probes for 679 rat mature miRNAs (miRBase version 16). In total, we were able to detect 267 mature miRNAs which were common to both nucleus and cytoplasm (Table S1). To check the reproducibility of microarray profiling, we compared data obtained from three different biological replicates of fractionations. All three biological replicates performed with cytoplasmic fractions exhibited similar expression patterns (Pearson's correlation coefficient, *r* = 0.93–95; Figure 1D). Likewise, all nuclear fractions showed comparable expression, albeit with a slightly lower correlation coefficient (*r* = 0.89–0.94; Figure 1D). Together, these data suggest that fractionations were reproducible and the microarray profiling procedure and normalization was appropriate. Interestingly, samples from nuclear and cytoplasmic compartments had a lower correlation coefficient (*r* = 0.74–0.85) between datasets (Figure 1D), implying that the miRNA expression profiles of nuclear and cytoplasmic compartments are distinct. The average expression of the majority of miRNAs was lower in the nucleus compared to the cytoplasm (Figure 1E), indicating that most of the miRNAs, as expected, are preferentially located in the cytoplasm.

To identify a set of nuclear-enriched miRNAs, we first calculated a nuclear enrichment score [NEnS; log_2_(NUC/CYT)] for all miRNAs, by log_2_ transforming the average ratio of nuclear/cytoplasmic signal intensity values (Table S1). The NEnS for individual miRNAs ranged from 10.14 to −3.50 with a median of −1.12, suggesting that on average miRNA expression in the cytoplasm is ~2-fold higher than in the nucleus. From a total of 267 miRNAs, 91 miRNAs (34.1%) displayed a statistically significant differential distribution between nuclear and cytoplasmic compartments (student's *t*-test, *p* < 0.05; Table S2). Among them, 87 (32.6%) miRNAs were preferentially found in the cytoplasm, and only 4 (1.5%; miR-133b^*^, miR-365^*^, miR-328a^*^, miR-92a) in the nucleus. Three of these miRNAs (miR-133b^*^, miR-365^*^, and miR-328^*^) were not previously reported to be expressed in neuronal cells. Therefore, to validate our results and to obtain a more comprehensive coverage of nuclear miRNAs, we decided to perform in addition deep sequencing of small RNAs from our fractionation experiment.

### Deep sequencing of small RNAs from nuclear and cytoplasmic fractions

In comparison to microarrays, deep sequencing-based profiling of small RNAs is more sensitive and allows to study the expression of miRNAs at nucleotide resolution. Furthermore, it allows to discriminate mature and precursor forms of miRNAs. Importantly, it also provides information about variable isoforms of miRNAs, so called isomiRs, and the nature of the associated nucleotide modifications. We used the Illumina-platform for deep-sequencing of small RNA libraries obtained from different compartments of rat primary cortical neurons (DIV7). To ascertain reproducibility of the results, we used two biological replicates for each cellular fraction. Moreover, to obtain a deep coverage of all possible isomiRs and to eliminate the effect of multiplexing artifacts which can result from different barcodes used in small RNA libraries, each of the small RNA libraries (2 nuclear and 2 cytoplasmic) were sequenced in individual lanes of one Illumina HiSeq flow cell.

**Figure 2:**
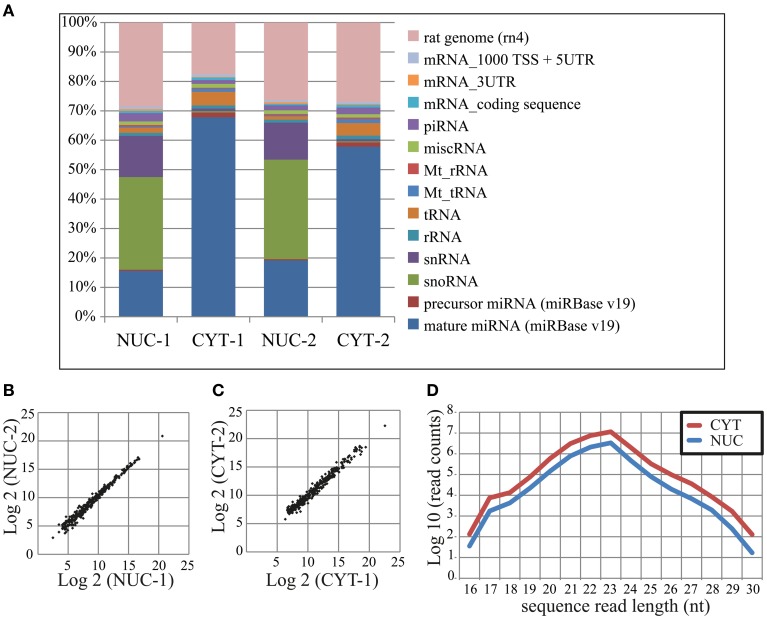
**Small RNA deep sequencing after nucleo-cytoplasmic fractionation of neurons. (A)** Proportional distribution of mapped sequencing read counts in the small RNA libraries. The total read counts were set to 100% for each small RNA library. **(B,C)** Scatterplots of log_2_ transformed read counts mapping to mature miRNAs from the nuclear **(B)** and cytoplasmic **(C)** fractions (two biological replicates each). **(D)** The sequence length distribution (x-axis) of reads mapping to mature miRNAs. The read counts (y-axis) were log_10_ transformed.

In total, we obtained ~62 and 66 million sequence read counts for nuclear and cytoplasmic fractions, respectively. After filtering reads according to the length (>15 nt), contamination (adapter sequences), quality and abundance (at least 2 identical reads per unique sequence), ~19 and 41 million “clean” reads, respectively, remained for further analysis. These reads were mapped to the publicly available rat RNA and genomic databases (rn4; Table 1; see Methods). To compare the abundance of read counts between two cellular fractions, a normalization according to the total number of mapped clean reads (nuclear—17,883,861; cytoplasmic—37,917,208) was performed. Interestingly, the number of normalized read counts from the nucleus matching to mature miRNA was 3–4-fold lower (depending on biological replicate) than in the cytoplasm (Figure 2A and Table S3). As expected, the nucleolar/nuclear small RNAs (snoRNA and snRNA) were highly enriched (63–89-fold and 16–17-fold, respectively) in the nuclear fraction; in contrast, cytoplasmic tRNAs were depleted (3-fold) in this fraction, again showing the purity of the cellular fractions used for sequencing. Furthermore, the expression of mature miRNAs in two biological replicates for each cellular fraction showed very high Pearson's correlation coefficient (nuclear, *r* = 0.99; cytoplasmic, *r* = 0.98), demonstrating a high reproducibility of the experiments (Figures 2B,C).

**Table 1:** **The summary of small RNA deep sequencing**.

	**Total NUC read counts**	**Total CYT read counts**	**Total read counts**
Total	62,931,202	65,995,793	128,926,995
disc^*^_<15 nt	7,163,839	7,572,786	14,736,625
disc_adapter only	870,091	1,389,233	2,259,324
disc_non-clipped	29,780,435	10,399,717	40,180,152
disc_qual_low	1,156,960	2,065,820	3,222,780
disc_one read per condition	4,968,122	3,930,256	8,898,378
mature miRNA (miRBase v19)	3,171,512	23,941,026	27,112,538
precursor miRNA (miRBase v19)	72,675	576,251	648,926
snoRNA	5,880,089	166,704	6,046,793
snRNA	2,352,776	300,101	2,652,877
rRNA	181,268	436,816	618,084
tRNA	247,157	1,688,530	1,935,687
Mt_tRNA	66,111	463,741	529,852
Mt_rRNA	87,352	147,452	234,804
miscRNA	218,657	466,009	684,666
piRNA	387,290	672,414	1,059,704
mRNA_coding sequence	104,619	330,211	434,830
mRNA_3UTR	96,117	92,012	188,129
mRNA_1000 TSS + 5UTR	138,915	289,055	427,970
rat genome (rn4)	4,879,323	8,346,886	13,226,209
mappable_all	17,883,861	37,917,208	55,801,069
not mapped	913,165	2,663,540	3,576,705

Numbers represent raw (non-normalized) read counts.

* disc_ discarded.

In total, we identified 335 miRNAs represented by at least 100 reads in one of the cellular compartments (Table S4). The size distribution of reads mapping to mature miRNAs peaks at 23 nt (Figure 2D), but not at 22 nt as was previously observed (Lee et al., 2010), probably owing to the high expression of miR-9 (49 and 44% of total reads in nuclear and cytoplasmic fractions, respectively). The overall distribution of read length in the nucleus was similar to the cytoplasm, but as mentioned above, with less total reads. The NEnS for individual miRNA ranged from 1.88 to −5.58 and the median was −2.08, when all detected miRNAs are considered (Table S4). Only two miRNAs, miR-143 and miR-126^*^ possessed a positive NEnS, indicating that these miRNAs are enriched in the nucleus according to deep sequencing. Since only two biological replicates were generated, the statistical parametric analysis was not applicable.

### Comparison of microarray and deep sequencing

**Figure 3:**
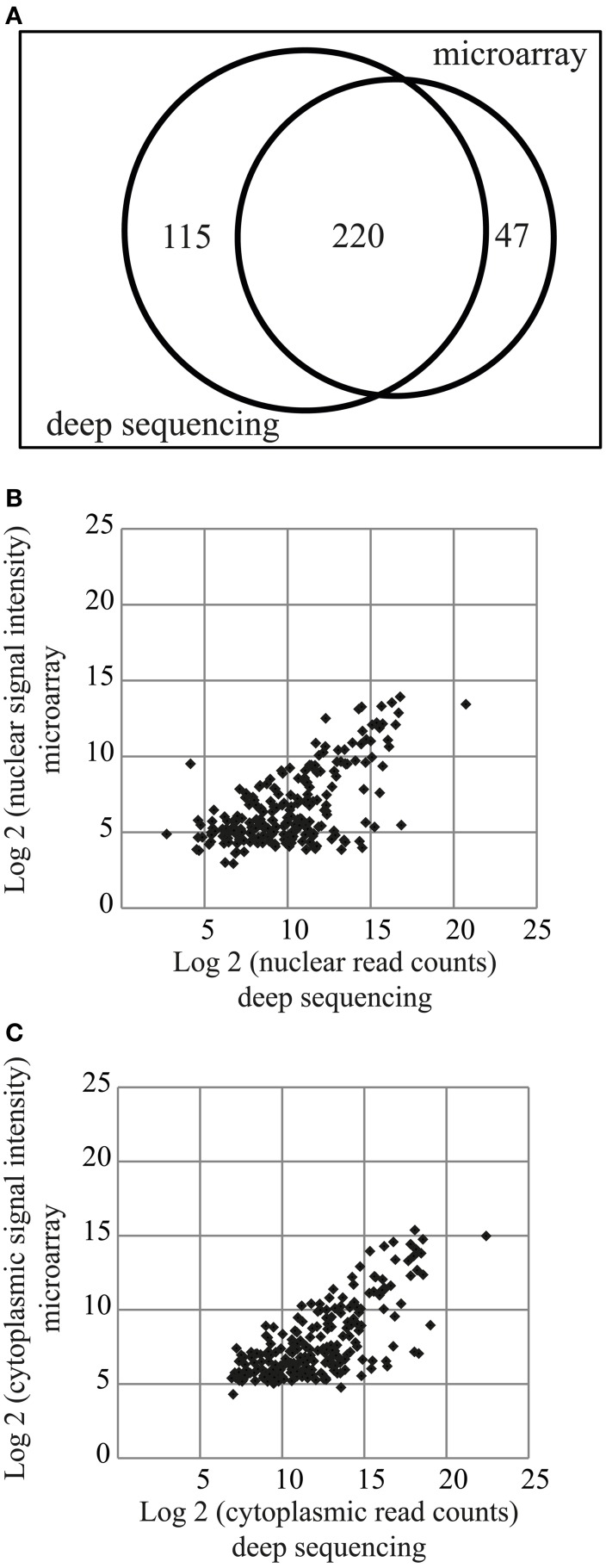
**Comparison of miRNA expression profiles obtained from miRNA microarrays and small RNA deep sequencing. (A)** Venn diagram illustrating miRNAs detected by the two different methods. 220 miRNAs were detected by both methods. **(B,C)** Scatterplot of log_2_ transformed signal intensity values (microarray, y-axis) and read counts (deep sequencing, x-axis) for miRNAs detected in the nuclear **(B)** or cytoplasmic **(C)** fractions.

Two hundred and twenty miRNAs were commonly detected by both microarray and deep sequencing methods, whereas 47 and 115 were specific for microarray or deep sequencing, respectively (Figure 3A). The comparison of miRNA expression [log_2_(signal intensity or read count)] data obtained with these two methods showed a Pearson's correlation coefficient of 0.63 and 0.71 for nuclear and cytoplasmic miRNAs, respectively (Figures 3B,C), suggesting that overall there is a correlation in the expression patterns between the datasets obtained from different methods. However, the correlation coefficient is much lower compared to biological replicates (Figures 1D, 2B,C). The major effect contributing to the difference between the data is probably that deep sequencing is more sensitive than microarray as illustrated in Figures 3B,C. The points corresponding to the low expressed miRNAs (data points log_2_ = ~5 on y-axis) according to microarray are shifted toward the right side of the x-axis, indicating that deep sequencing, in contrast to microarray, can effectively detect and discriminate between low expressed miRNAs. This is even more apparent in the nuclear fraction (Figure 3B). Since the NEnS of miRNAs is calculated from the log_2_ transformed ratio of nuclear and cytoplasmic expression levels (signal intensity or read counts), a cross-platform difference in detection efficacy of miRNA expression might result in rather different NEnS for the same miRNA depending on the method. Indeed, NEnS scores for miRNAs obtained from microarray and deep-sequencing experiments showed no correlation (Pearson's correlation coefficient, *r* < 0.1, data not shown), and therefore statistical parametric analysis was not applicable. Hence we sought to employ alternative statistical methods to compare datasets from microarray and deep sequencing.

**Figure 4:**
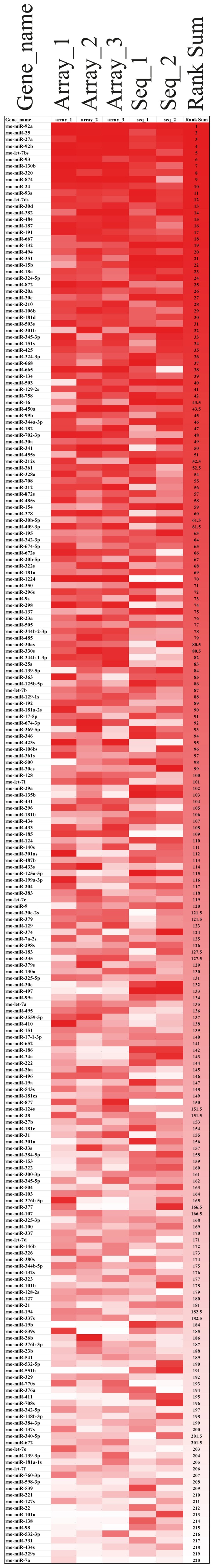
**Alignment of miRNAs according to their miRNA nuclear enrichment scores (NEnS) obtained with microarrays and deep sequencing**. MiRNAs were ranked from high (red) to low (white) NEnS for each experiment separately (array_1, _2, _3, seq_1, _2), and then the average ranking was calculated and arranged in descending order based on the Rank Sum method (Laing and Smith, 2010).

Rank-based non-parametric statistics employs the ranks instead of actual expression levels to identify differentially expressed genes (Hong et al., 2006). Therefore, this type of analysis is less sensitive to “noise” between the data obtained using different high-throughput platforms, such as microarray (Hong and Breitling, 2008) and deep sequencing (Llorens et al., 2013), and allows determining the genes, in our case miRNAs, which are consistently high-ranked in data obtained using different methods. We used the Rank Sum method to identify miRNAs, which possess consistently high (higher than other miRNAs) NEnS ranking in both microarray and deep sequencing, and therefore potentially might be enriched in the nucleus (Hong et al., 2006; Laing and Smith, 2010). For this analysis miRNAs that were detected by both platforms (220) were considered. As illustrated in the rank-based heatmap, miRNAs are color-coded from red to white in descending rank order for each biological replicate separately (Figure 4, columns 1–5 and Table S5) and together (Figure 4, column 6 and Table S5). Despite some differences in the ranking, the overall ranking of miRNAs is highly similar not only between different biological replicates, but also between different technological platforms. After applying Benjamini-Hochberg false discovery rate (FDR) of 0.05 for multiple testing (Benjamini et al., 2001), we identified 8 miRNAs, which were significantly higher ranked among the biological replicate experiments of microarray and deep sequencing (Table 2), suggesting that these miRNA might be preferentially localized to the neuronal nuclei compared to the vast majority of miRNAs. Importantly, the synaptic miR-7a and miR-138 (Siegel et al., 2009) were among the 10 most low ranked miRNAs (i.e., cytoplasmic; Table 2), suggesting that the rank-based analysis method is able to faithfully detect differences in the intracellular distribution of miRNAs.

**Table 2:** **Top 10 high-ranked and top 10 low-ranked miRNAs according to Rank Sum method**.

**miRNA_name**	**Rank sum rank**
rno-miR-92a	1
rno-miR-25	2
rno-miR-27a	3
rno-miR-92b	4
rno-let-7b^*^	5
rno-miR-93	6
rno-miR-130b	7
rno-miR-320	8
rno-miR-874	9
rno-miR-24	10
rno-miR-127^*^	211
rno-miR-22	212
rno-miR-101a	213
rno-miR-138	214
rno-miR-98	215
rno-miR-532-3p	216
rno-miR-331	217
rno-miR-434^*^	218
rno-miR-329^*^	219
rno-miR-7a	220

### Validation of nuclear-enriched miRNA candidates identified by profiling approaches

**Figure 5:**
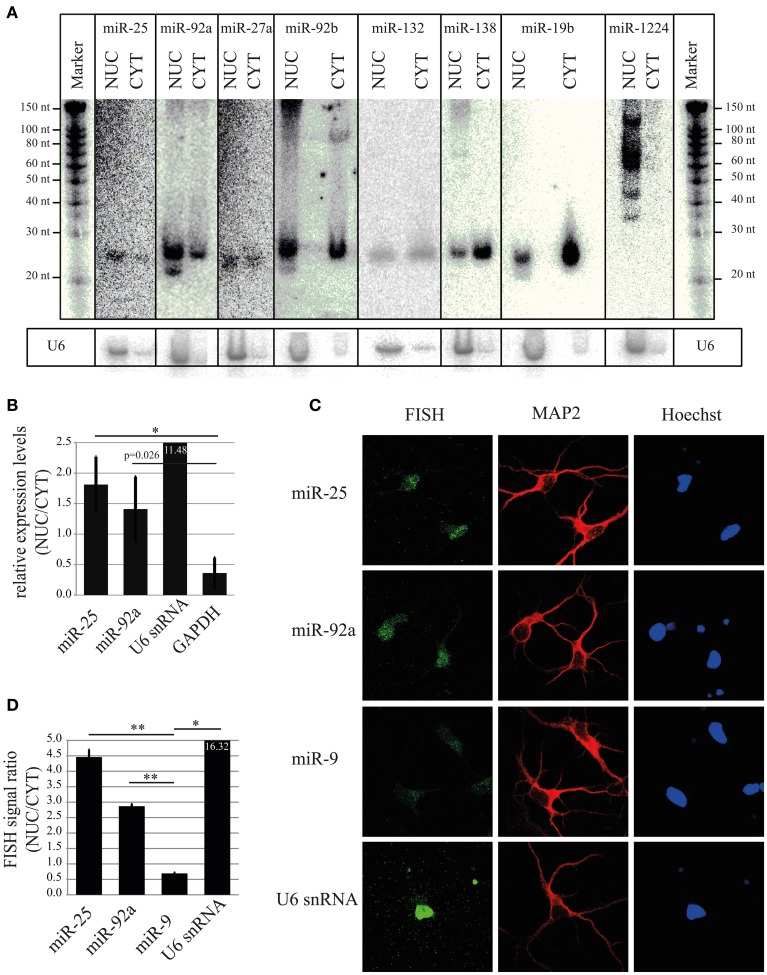
**Validation of nuclear expression for selected miRNA candidates. (A)** Northern blot analysis of nuclear-enriched (miR-25, miR-92a, miR-27a, miR-92b) and -depleted miRNAs (miR-138, miR-19b). As a control for the fractionation efficacy, U6 snRNA was probed. **(B)** qRT-PCR analysis of nuclear-enriched miRNAs (miR-25 and miR-92a). The fold enrichment (y-axis) of miRNAs and marker genes (U6 snRNA and GAPDH) in the nucleus was calculated by the 2^−dCt^ [2^−(NUC Ct−CYT Ct)^] method. Bar plots show mean ± *SD* (*n* = 3). Statistical significance was determined using Student's *t*-test with Bonferroni correction (^*^p < 0.05). **(C)** Subcellular localization of the indicated miRNAs at DIV5 hippocampal neurons as assessed by fluorescent *in situ* hybridization assay (FISH) using Digoxigenin (DIG) labeled miRCURY LNA probes (green). FISH for U6 was used as a positive control for nuclear localization. MAP2 protein was used to identify neurons (red). Hoechst counterstain was used to label nuclei (blue). **(D)** Quantification of nuclear localization from FISH experiment presented in **(C)**. Signal intensities within the nucleus and cytoplasm were determined with ImageJ. The ratios of nuclear/cytoplasmic signal intensities are shown as an indicator for nuclear enrichment. Bar plots show mean ± *SD* (*n* = 2; 10 cells per condition of a single experiment). Statistical significance was determined using Student's *t*-test with Bonferroni correction (^*^p < 0.05; ^**^p < 0.01).

To validate results obtained using microarray and deep sequencing with a Rank Sum analysis, we decided to perform Northern blot, which allows size-separation and visualization of miRNAs with different sizes, including mature miRNA. As shown in Figure 5A (and Figures S1B,C), the mature form of four highly ranked miRNAs (miR-92a, miR-25, miR-27a, and miR-92b) was higher or equally expressed in the nuclear fraction compared to the cytoplasm. In contrast, a low–ranked miRNA, miR-138 (rank = 214), showed the opposite expression pattern. Interestingly, if only one method, for instance deep sequencing, is taken into account to calculate nuclear-enrichment, then the ranks for miR-92a, miR-25, miR-27a, and miR-92b are 7, 31, 28 and 34, respectively (Table S5). According to the same method miR-132 is ranked 3, implying that this miRNA should be more nuclear enriched than the other four. However, miR-132 possessed slightly less signal in the nucleus compared to the cytoplasm by Northern, which is more in line with the ranking (rank = 19) when both methods (Rank Sum) are taken into account (Table S5). A similar rank correction is observed for miR-19b (deep seq rank = 49; Rank Sum rank = 184), for which Northern showed a similar depletion of signal in the nucleus compared to the cytoplasm as miR-138 (Figure 5A). Likewise, the miRNAs highly ranked using only microarray data are either not detected by deep sequencing (miR-133b^*^, miR-365^*^, and miR-328^*^) or their ranking (Rank Sum) is decreased considerably (miR-1224; Figure 5A and Table S5). This is in line with the Northern blot data which suggests that the nuclear signal for these miRNAs is possibly originating from by-products of pre-mRNA splicing or non-coding RNA transcription, but not from the mature miRNA (Figure 5A). Taken together, these results confirm that some of the highly ranked miRNAs (miR-92a, miR-25, miR-27a, and miR-92b) are indeed enriched in the nucleus and also indicates the robustness of the rank-based statistical analysis to identify nuclear-enriched or -depleted miRNAs.

In addition to Northern blot assay, we further validated nuclear enrichment of the two top candidate miRNAs, miR-25 and miR-92a using TaqMan qRT-PCR. In agreement with results from Northern blot, miR-25 and miR-92a showed a significant nuclear enrichment compared to GAPDH, with a NUC/CYT fold change of 1.81 and 1.41, respectively (Figure 5B). As expected, the nuclear marker gene U6 was enriched (11.48) in the nucleus, whereas the cytoplasmic marker gene GAPDH was depleted (0.36), once more demonstrating that the cellular fractionation protocol was efficient in separating nuclei and cytoplasm.

For all experiments so far, we used total RNA from nuclear and cytoplasmic compartments. This RNA was obtained from a biochemical fractionation method that relies on differential centrifugation. With this method, it is difficult to achieve complete separation of compartments, and therefore the obtained results might not entirely reflect the natural distribution of miRNAs in intact neurons. Moreover, biochemical preparations likely contain a mixture of RNA from different cell types, e.g., neurons and glia. Thus, we performed in addition fluorescent *in situ* hybridization (FISH) with LNA probes to precisely determine localization of nuclear-enriched miRNAs (miR-25 and miR-92a) in intact primary rat hippocampal neurons (DIV5) at the single cell level (Figure 5C). After application of a FISH probe against miR-25 and miR-92a a stronger fluorescent signal in the neuronal nucleus compared to the cytoplasm was observed, indicating that these miRNAs are preferentially localized in the nucleus of intact neurons. Conversely, the cells hybridized with a probe against miR-9 (Rank Sum rank = 120) displayed a stronger fluorescent signal in the cytoplasm compared to the nucleus. Accordingly, quantification of FISH signal from many cells revealed that the ratio between nuclear and cytoplasmic signals for miR-25 and miR-92a was significantly higher (*p* = 0.002 and 0.0006, respectively, Student's *t*-test) than miR-9 (Figure 5D). Taken together, our results from Northern, qRT-PCR and FISH strongly suggest that miR-25 and miR-92a are enriched in the nucleus of post-mitotic primary rat neurons.

### Developmental expression levels of miRNAs and their nuclear enrichment

**Figure 6:**
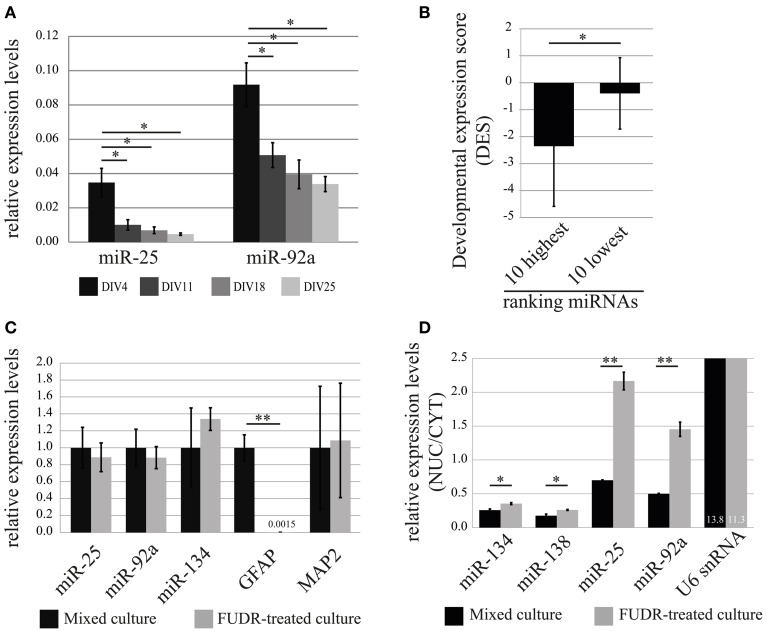
**Developmental stage and cell-type-specific expression of the nuclear-enriched miRNAs, miR-25 and miR-92a. (A)** Relative expression (normalized to U6 snRNA) levels of miR-25 and miR-92a during *in vitro* development of primary cortical neurons was determined by qRT-PCR analysis. Bar plots show mean ± *SD* (*n* = 2). Statistical significance was determined using Student's *t*-test with Bonferroni correction (^*^, *p* < 0.05). **(B)** Developmental expression score (DES; log_2_(P3/E10) from (Yao et al., 2012); y-axis) comparison of 10 highest and lowest ranked miRNAs. Error bars represent standard deviation from the mean DES within each group. Statistical significance was determined using Student's *t*-test (*p* = 0.028). **(C)** Expression of miR-25 and miR-92a in mixed cultures and neuronal-enriched cultures (FUDR-treated). The relative expression levels of indicated RNAs were obtained by the ddCt method. RNA levels in mixed cultures were arbitrarily set to 1. Bar plots show mean ± *SD* (*n* = 3). *SD* for mixed culture condition was determined after normalization to an internal control RNA (U6 snRNA). Statistical significance was determined based on U6 snRNA normalized values using Student's *t*-test with Bonferroni correction (^**^p < 0.01). **(D)** Nuclear-enrichment of miRNA expression in mixed and neuron-enriched (FUDR-treated) cultures. The expression level of miRNAs was determined using qRT-PCR analysis with TaqMan microRNA assay. Bar plots show mean ± *SD* (*n* = 2). Statistical significance was determined using Student's *t*-test with Bonferroni correction (^*^p < 0.05; ^**^p < 0.01).

In order to obtain a first indication at which developmental stage miR-25 and miR-92a might function in neurons, we performed a developmental time-course experiment, quantifying the relative expression levels of mature miRNAs at 4, 11, 18, 25 DIV in cortical neurons using qRT-PCR (Figure 6A). The expression levels of both miR-25 and miR-92a were significantly declining with the progress of neuronal development, whereby the decrease in expression of miR-25 was more pronounced compared to miR-92a. At the end of the developmental time-course (DIV25), expression levels of miR-25 and miR-92a were reduced by 80% and 60%, respectively, compared to DIV4. Taken together, our results indicate that expression of miR-25 and miR-92a is down-regulated during post-mitotic neuronal development. However, since measurements were started at DIV4, we cannot rule out that the peak of expression for these miRNAs is actually even earlier in development.

Based on our observations, we tested the hypothesis that developmental down-regulation might be a common feature of nuclear-enriched miRNAs. We therefore calculated a developmental expression score (DES; see Methods) for each miRNA present in our ranking list based on a recent study which reported genome-wide miRNA expression profiles during development of the rat cortex *in vivo* (Yao et al., 2012). A negative DES would hereby indicate that the expression level of the respective miRNA is down-regulated during rat cortex development. Indeed, we observed a trend toward an increase of the average DES from high (nuclear-enriched) to low (cytoplasmic-enriched) ranking miRNAs, suggesting that down-regulation during rat cerebral cortex development is a common feature of nuclear-enriched miRNAs (Figure S1D and Table S6). Accordingly, the DES of two extreme groups consisting of the 10 highest and lowest ranked miRNAs (hence, the most reliable in terms of nucleo-cytoplasmic localization), differ significantly (*p* = 0.028; Student's *t*-test) with an average DES of −2.35 and −0.39, respectively (Figure 6B).

Taken together, these findings suggest that nuclear-enriched miRNAs in general might be expressed at early stages of neuronal development and that their decline in expression levels during development correlates with nuclear enrichment.

### miR-25 and miR-92a are specifically enriched in neuronal nuclei, but not in glia

Results from recent publications suggest that miR-25 and miR-92a might be preferentially expressed in glia compared to neurons (Jovicic et al., 2013). In order to investigate the contribution of glial cells to the expression of miR-25 and miR-92a in our primary cortical neuronal cultures, we further decided to test expression levels of these miRNAs in glia-depleted and glia-enriched neuronal cultures. Primary cortical neurons prepared with our standard protocol contain a substantial amount of proliferating glial cells (10–20% of total cells at DIV7, data not shown). We therefore considered the possibility that glia-derived miRNAs could significantly contribute to the results concerning nuclear-enrichment of miRNAs in neurons. To obtain glia-depleted culture, we cultured cells in the presence of a potent inhibitor of cell proliferation, 2′-Deoxy-5-fluorouridine (FUDR), and relative expression levels of miRNAs were assessed by qRT-PCR. Depletion of glial cells in our cultures was verified by the quantification of the astrocytic marker gene glial fibrillary acidic protein (GFAP), which was almost completely absent in FUDR-treated cultures (Figures 6C, S2A). The expression of neuronal marker genes, miR-134 and MAP2 were not significantly affected by FUDR-treatment, suggesting that the overall contribution of RNA from glial cells to the total RNA in our mixed cultures is small. Importantly, the nuclear-enriched miRNAs, miR-25 and miR-92a, in contrast to GFAP, were only slightly reduced in FUDR-treated cultures, showing that the expression of these miRNAs in our mixed cultures is predominantly derived from neurons, with a small contribution from glia.

Experiments carried out on glia-depleted cultures indicate that the overall contribution of glial cells to the expression of nuclear-enriched miRNAs in our mixed cultures is small. However, they do not rule out that the expression of these miRNAs in an individual glial cell is in fact higher compared to that in an individual neuron. We therefore established a culture protocol that strongly favors the growth of glial cells (approximately 50–60% are glial cells; data not shown). In glia-enriched cultures, we could detect higher expression of GFAP, and lower expression of miR-134 and MAP2 compared to mixed culture, suggesting that these culture conditions indeed favored the growth of glial cells (Figure S2B). Interestingly, miR-25 and miR-92a displayed a 2.2 and 1.5-fold, respectively, higher expression in glia-enriched cultures compared to mixed culture, suggesting that expression of these miRNAs is in fact higher in individual glial cells compared to neurons.

Finally, we wanted to compare nuclear enrichment in glial cells and neurons. For this we fractionated mixed and FUDR-treated cultures in nuclei and cytoplasm, and then measured RNA expression by TaqMan qRT-PCR (Figure 6D). Interestingly, the nuclear enrichment of both miR-25 and miR-92a was on average 3-fold higher in FUDR-treated cultures compared to mixed cultures. In contrast there was no significant change in the nuclear enrichment of miR-134, miR-138 and U6 snRNA, demonstrating the specificity of the assay. These results suggest that miR-25 and miR-92a are specifically enriched in the nucleus of neurons, but not glial cells, where they might instead preferentially localize to the cytoplasm.

In summary, although miR-25 and miR-92a are clearly expressed in glial cells, the major contributors to their expression in mixed cultures are neurons. Furthermore, nuclear-enrichment of these miRNAs is a specific feature of neurons. These results are consistent with FISH and suggest a specific function of miR-25 and miR-92a in the nucleus of post-mitotic neurons.

### Inspection of nuclear miRNAs for common sequence characteristics

Since localization of RNAs to distinct cellular compartments is known to be dependent on specific cis-acting sequences (Jambhekar and Derisi, 2007), we decided to search for common cis-acting elements that might target miRNAs to the neuronal nucleus. In this regard, it was shown that a 3′ hexanucleotide motif (AGUGUU) is sufficient to direct miR-29b into the nucleus of HeLa cells (Hwang et al., 2007; Jeffries et al., 2010). Furthermore, it was reported that in human neural progenitor cells, 7 out of 21 miRNAs with preferential nuclear localization possess an ASUS (S = G or C; this motif is also included in the aforementioned miR-29b) motif within the last 3′ 9 nt (Jeffries et al., 2011). However, the ASUS motif was neither enriched nor depleted in the last 3′ 10 nt of two extreme groups consisting of the top 20 high-ranked and top 20 low-ranked miRNAs (Table S5), suggesting that in contrast to the results from non-neuronal systems (Jeffries et al., 2011) the ASUS motif does not function as a nuclear localization signal in neurons.

Furthermore, it was reported that miRNAs which have the same seed sequence and a similar composition of the nine 3′-terminal nt, are likely to be enriched in the same cellular compartment [nuclear or cytoplasmic; (Jeffries et al., 2011)]. In agreement, we found that three members of a miRNA family [miR-92a (rank = 1), miR-25 (rank = 2) and miR-92b (rank = 4)] which in addition to the seed share a common 3′-terminus are high ranked, whereas another member of the same family with a different 3′-terminus [miR-363 (rank = 85); Figure S3] is low ranked. However, some other miRNA pairs with similar nucleotide composition, such as miR-27a (rank = 3)/miR-27b (rank = 153) or miR-130b (rank = 7)/miR-130a (rank = 130) were not ranked together (Figure S3), suggesting that having the same seed together with a similar 3′-terminus alone is not sufficient to confer nuclear enrichment in neurons. Therefore, in addition to the similarity of seed and 3′-terminus, other sequence elements might also be important for nuclear localization of miRNAs.

A closer inspection of the nuclear rank list revealed that highly ranked miRNAs have a tendency to contain a guanine (G) at the 3′-terminus, whereas low ranking miRNAs often end with a uridine (U; Figure S4). However, a statistical analysis of the two extreme groups (top 10 high-ranked and 10 low-ranked miRNAs) did not show a significant difference (data not shown). We further investigated if the high-ranked miRNAs share any other sequence motifs. However, none of the online available multiple alignment and motif finding tools (ClustalW, MEME, LocARNA, Gibbs motif sampler) found any over-represented motifs among nuclear-enriched miRNAs (data not shown).

Taken together, bioinformatic inspection of miRNAs for putative cis-acting sequence elements revealed that in contrast to previously published results, the ASUS motif at the 3′ region of miRNAs is evenly distributed through-out our ranking list, suggesting that in neurons this motif is unlikely to participate in nuclear localization. Moreover, a similar nucleotide composition alone is not a faithful predictor of nuclear enrichment, implying that nuclear localization of miRNAs probably involves multiple, sequence- and structure dependent mechanisms.

### IsomiRs with a 3′-terminal guanine preferentially localize to a nucleus

**Figure 7:**
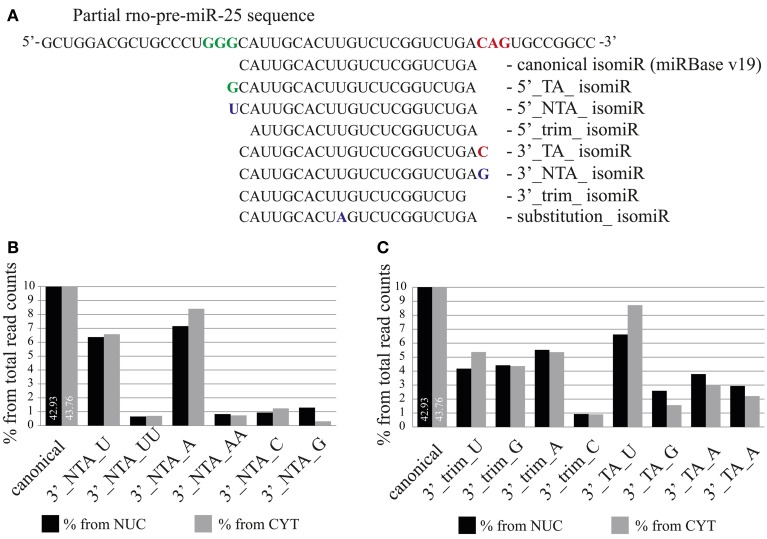
**The distribution of isomiRs between nuclear and cytoplasmic fractions of neurons. (A)** Definition of different isomiR species. For illustration, miR-25 isomiRs aligned to the pre-mir-25 sequence are shown (TA, templated addition; NTA, non-templated addition; trim, trimmed). **(B,C)** Proportion of specific isomiRs from the total sequence reads which mapped to miRNAs in the nuclear (black) and the cytoplasmic (gray) fractions. **(B)** Proportion of isomiRs with specific 3′ non-templated additions. **(C)** Proportion of isomiRs with 3′ trimmed and templated additions. The respective added or trimmed nucleotides are indicated for specific isomiRs.

Analysis of mature miRNA localization did not reveal the presence of a common sequence element responsible for nuclear accumulation. However, a slight trend for the presence of a 3′-terminal guanine was observed (Figure S4). We therefore took advantage of the high sequence coverage of our deep sequencing datasets, which allows the analysis of individual isomiRs, even those expressed at low levels. IsomiRs are variants of canonical miRNAs containing 5′ and 3′-end variations, which either result from a variability in the cleavage of Drosha and Dicer [templated nucleotide addition (TA) or trimming] or from non-templated nucleotide addition (NTA; Figure 7A) catalyzed by nucleotidyltransferases.

**Figure 8:**
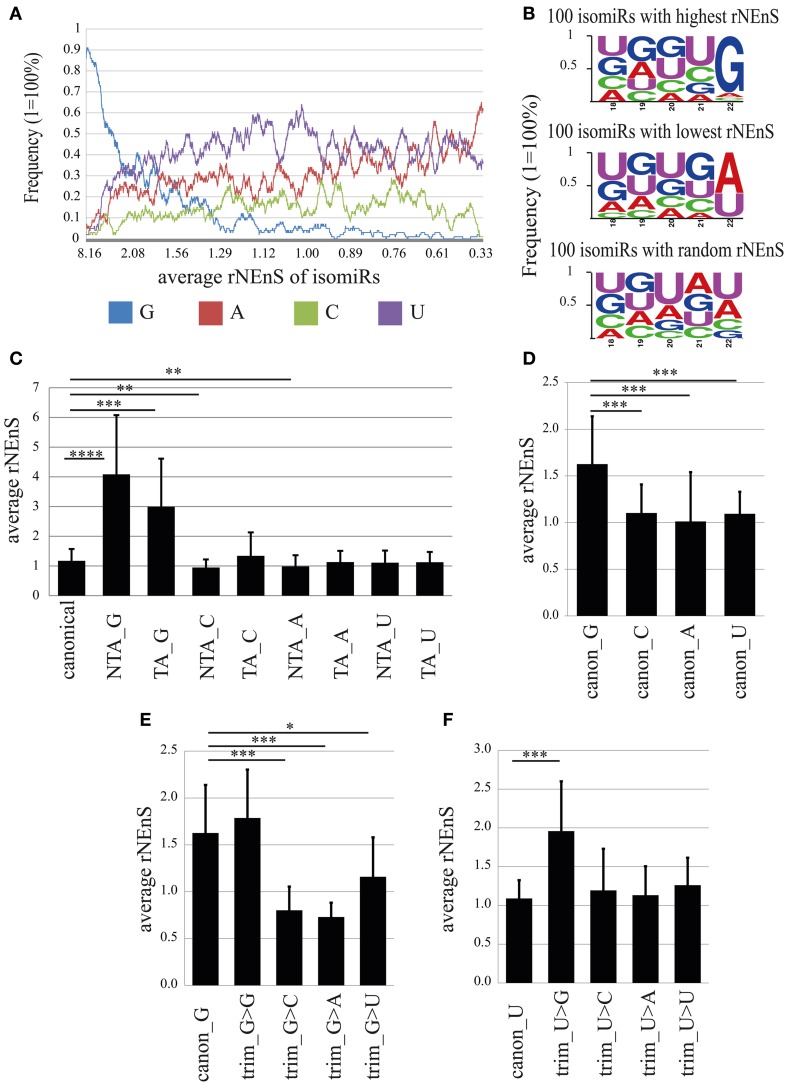
**The impact of the 3′-terminal nucleotide on nuclear localization. (A)** Frequency of different nucleotides at the 3′-terminus of isomiRs depending on the relative nuclear enrichment score (rNEnS). Nucleotide frequency (y-axis) was calculated using moving window technique, where window length was set as 100 and the average frequency values were calculated by moving the window with one step at a time from isomiRs (in total 4661) possessing high to low rNEnS. In the x-axis, the average rNEnS of isomiRs using moving window technique with the same parameters as above is depicted. **(B)** Frequency of different nucleotides in the 3′-terminal 5 nts of 100 isomiRs with highest (upper panel), lowest (middle panel) and random rNEnS (lower panel). **(C)** Impact of 3′ non-templated (NTA) and templated additions (TA) on the relative nuclear localization. Bar plots show mean ± *SD* [*n* = from 31 (TA_G) to 306 (canonical)]. Statistical significance was determined using Welch′s *t*-test (unequal variance) with Bonferroni correction (^*^p < 0.05; ^**^p < 0.001; ^***^p < 0.00001; ^****^p < 1.0-E10). **(D)** rNEnS for canonical isomiRs containing different 3′-terminal nucleotides. Bar plots show mean ± *SD* (canon_G, *n* = 57; canon_C, *n* = 62; canon_A, *n* = 75; canon_U, *n* = 112). Statistical significance was determined using Welch′s *t*-test (unequal variance) with Bonferroni correction (^*^p < 0.05; ^**^p < 0.001; ^***^p < 1.0-E7). **(E,F)** Impact of trimming on the nuclear localization of isomiRs. **(E)** rNEnS for canonical isomiR_G that underwent 3′ trimming, thereby exposing the indicated nucleotide. Bar plots show mean ± *SD* (canon_G, *n* = 57; trim_G>G, *n* = 12; trim_G>C, *n* = 14; trim_G>A, *n* = 17; trim_G>U, *n* = 9). Statistical significance was determined using Welch′s *t*-test (unequal variance) with Bonferroni correction (^*^p < 0.05; ^**^p < 0.001; ^***^p < 1.0-E5). **(F)** rNEnS for canonical isomiR_U that underwent 3′ trimming, thereby exposing the indicated nucleotide. Bar plots show mean ± *SD* (canon_U, *n* = 112; trim_U>G, *n* = 32; trim_U>C, *n* = 29; trim_U>A, *n* = 10; trim_U>U, *n* = 20). Statistical significance was determined using Welch′s *t*-test (unequal variance) with Bonferroni correction (^*^p < 0.05; ^**^p < 0.001; ^***^p < 1.0–E5).

As previously reported by several groups (Lee et al., 2010; Zhou et al., 2012), we also found that the abundance of isomiR types was miRNA specific. For instance, the sequence reads for the canonical form (miRBase v19) and for 3′-terminal single nucleotide templated addition (TA_1) forms of miR-138 were equally abundant and together comprised more than 50% (cytoplasm) −70% (nucleus) of the total reads for this miRNA (Figure S5). In contrast, for miR-25 and miR-92a the canonical and TA_1 forms, respectively, were overrepresented by 70% in both cellular compartments (Figure S5). In order to determine the overall abundance of specific isomiRs in the nuclear and the cytoplasmic fractions we calculated the percentage of the isomiRs, considering the entire nuclear or cytoplasmic sample. MiR-9 was excluded from the analysis, since the read counts for this miRNA comprise 49 and 44% of the total reads in nuclear and cytoplasmic fractions, respectively, and therefore might change the overall isomiR profile considerably. Our analysis showed that canonical miRNAs added to 42.93 and 43.76% of the total nuclear and cytoplasmic sequence reads, respectively (Figure 7B). As previously reported (Wyman et al., 2011; Zhou et al., 2012), the most abundant form of isomiRs were non-templated additions of single adenine (7.16%-nucleus; 8.40%-cytoplasm) and uracil (6.37%-nucleus; 6.58%-cytoplasm) nt, both of which were slightly overrepresented in the cytoplasm. Interestingly, an overall relatively rare non-templated addition of a single guanine (NTA_G) was 4-fold higher in the nucleus (1.30%) compared to the cytoplasm (0.31%). Furthermore, isomiRs with templated addition of a single guanine (TA_G) were also more prominent in the nuclear (2.59%) than in the cytoplasmic (1.56%) fraction (Figure 7C). This calculation is based on the abundance of the sequence reads, and distributions might be skewed by a few isomiRs of very abundant miRNAs. We note that the top 15 highly expressed miRNAs together account for 77.5% (cytoplasm) of all sequencing reads. In order to avoid the influence of the read counts, we first calculated a relative nuclear enrichment score (rNEnS; % of the nuclear fraction/% of the cytoplasmic fraction for a respective miRNA; see Methods) of isomiRs and then quantified the type (A, U, G, C) and occurrence of nt at the 3′-terminus of each unique sequence. Although we will be not able to differentiate the source of the last nucleotide variation (trimming, templated or non-templated additions) with this analysis, we can obtain an estimate how the 3′-terminal nucleotide influences nuclear localization. Strikingly, guanine at the 3′-terminus of isomiRs with a high rNEnS was strongly overrepresented compared other nt (Figures 8A,B). However, as the rNEnS declined guanine at the 3′-terminus became less frequent, whereas other nt (A, U, and C) were now more prominent. A closer inspection of specific miRNA isomiRs (Figure S6) confirmed our observation that isomiRs with a high rNEnS tend to possess a guanine nucleotide at their 3′-terminus. This data implies that 3′ guanine could promote nuclear accumulation of isomiRs.

In order to determine whether 3′-terminal G (canonical, trimmed, NTA, or TA), independent of the remaining sequence, has an impact on nuclear localization, we calculated rNEnS for different isomiRs (Figures 8C–F). Strikingly, the average rNEnS for non-templated (NTA_G; 4.08) and templated guanine added (TA_G; 2.99) isomiRs were significantly higher (*p* = 4.2E-13 and 6.8E-07, respectively; welch *t*-test) than the average rNEnS of all canonical isomiRs (1.17), irrespective of the 3′-terminal nucleotide (Figure 8C). In contrast, the average rNEnS for NTA_C (0.95; *p* = 4.9E-06) and NTA_A (0.99; *p* = 2.6E-06) was lower than for all canonical isomiRs. These results suggest either the possibility of targeted guanylation in the nucleus or enhanced localization to the nucleus of isomiRs already possessing a 3′ guanine due to NTA_G or TA_G. We next calculated the impact of the 3′-terminal nucleotide in canonical isomiRs. Surprisingly, the average rNEnS for canonical isomiR_Gs (1.63) was also significantly higher than canonical isomiR_C (1.1; *p* = 1.8E-09), _A (1.0; *p* = 4.3E-12) and _U (1.1; *p* = 1.6E-10) (Figure 8D). Furthermore, one nucleotide trimming of canonical isomiR_C, _A and _U and thereby exposing a guanine, but not other nt at the 3′-terminus, increased significantly the rNEnS for these isomiRs (Trim_N>G; N = A, C, or U; Figures 8F, S7). Conversely, one nucleotide trimming of canonical isomiR_G and thereby exposing nt other than guanine at the 3′-terminus significantly decreased the rNEnS (Figure 8E). Together, these results strongly argue that the guanine nucleotide at the 3′-terminus *per se* might lead to a preferential nuclear localization of isomiRs.

Taken together, we found that isomiRs possessing a 3′-terminal guanine nucleotide show preferential localization to the nucleus. The “origin” of this 3′-terminal guanine (NTA, TA, trimmed, or canonical) further influences the extent of nuclear localization.

## Discussion

It is increasingly recognized that miRNAs, in addition to their well described role as post-transcriptional regulators of mRNA translation/stability in the cytoplasm, are also involved in transcriptional (Kim et al., 2008; Place et al., 2008; Benhamed et al., 2012) and post-transcriptional (Hansen et al., 2011; Tang et al., 2012) regulatory processes in the nuclei of proliferating cells. However, a function of miRNAs in the nucleus of post-mitotic cells has not been described. As a first step in the determination of a putative nuclear role of miRNAs, we assessed the complete miRNA nuclear-enrichment profile and sequence-specific requirements that might aid (or be responsible for) the nuclear localization of miRNAs (and their isomiRs) in rat post-mitotic primary neurons.

In this study, we applied the two most common high-throughput profiling technologies, microarray and deep sequencing, to identify the nucleo-cytoplasmic distribution of miRNAs. In line with previous reports (Liao et al., 2010; Jeffries et al., 2011) we also detected the expression of almost all cytoplasmic miRNA counterparts in the nucleus. However, unlike these previous publications, our results from both profiling methods suggested that the majority of miRNAs are enriched in the cytoplasm and only a small subset in the nucleus. The discrepancy between these earlier findings and our current observations might be due to different cell types [cancer cell line (Liao et al., 2010), neural progenitor cells (Jeffries et al., 2011), post-mitotic neurons (this study)] used in these studies. It is also possible that the data normalization (Jeffries et al., 2011) and the power (Liao et al., 2010) of statistical analysis might have contributed. The normalization method performed by Jeffries et al. (2011) assumes that only a minority of genes are differentially expressed between conditions (i.e., normalized to the mean/median expression value of all miRNAs detected within the single replicate experiment). Without *a priori* knowledge of nucleo-cytoplasmic distribution of miRNAs, this type of data normalization might not be appropriate to measure the absolute differences in the expression levels of miRNAs (although this does not affect the nuclear-enrichment ranking between miRNAs) in nuclear and cytoplasmic compartments, since it equalizes otherwise initially different expression profiles in these compartments. To overcome this limitation and to measure absolute miRNA expression levels we therefore used exogenous controls, spike-in oligoribonucleotides (microarray) and total RNA/genomic mapped reads (deep sequencing) for cross-compartmental normalization of miRNA expression. Liao and colleagues used only one biological replicate for deep sequencing, thereby lacking any statistical power. In contrast, we used five biological replicates (3 for microarray and 2 for deep sequencing) and identified nuclear-enriched miRNAs in neurons based on the non-parametric Rank Sum method. Interestingly, the application of both microarray and deep sequencing gives more reliable results than each method separately with regard to the identity of nuclear-enriched miRNAs. Based on further validation results (Northern blot) of nuclear- and cytoplasmic-enriched miRNAs we presume that at most 5% of the 220 miRNAs analyzed by both profiling methods are truly nuclear-enriched miRNAs, although additional experiments are required to validate the expression of more high ranked miRNA candidates.

In addition to the overall distribution of miRNAs between the nuclear and cytoplasmic compartments, there is also little overlap regarding the identity of nuclear-enriched miRNAs among our and earlier reports, which might be accounted for by cell-type and differentiation stage (proliferating vs. non-proliferating) specific differences in miRNA expression. For example, members of the miR-25 family (miR-25 and miR-92a) are found to be preferentially localized in the cytoplasm of human neural stem cells (Jeffries et al., 2011), whereas we found that these miRNAs are enriched in the nuclei of post-mitotic neurons. Furthermore, the miR-25 family members are overexpressed in different cancer types (Kim et al., 2009; Li et al., 2009), and are implicated in the inhibition of pro-apoptotic and anti-proliferative genes such as tumor protein 53 (Kumar et al., 2011) and BCL-2 family protein BIM; (Tsuchida et al., 2011; Zhang et al., 2012), a regulation which presumably occurs in the cytoplasm. Therefore, it is likely that in the early stages of neural development (e.g., in neural progenitors), miR-25 family members localize to the cytoplasm and are involved in the post-transcriptional regulation of proteins involved in the control of cell cycle and proliferation. Indeed, overexpression of miR-25 increased the proliferation of mouse neural stem/progenitor cells [NSPC; Brett et al., 2011] and also induced re-entry into mitosis of post-mitotic neurons from zebrafish spinal cord by directly inhibiting the expression of p57 cell-cycle inhibitor (CDKN1C) (Rodriguez-Aznar et al., 2013). Likewise, miR-25 family members might suppress the expression of neuronal phenotype promoting genes in the cytoplasm of glial cells, since the gene ontology (GO) terms, such as neuron development and differentiation, are enriched in the predicted mRNA targets for these miRNAs. In contrast, we found that miR-25 and miR-92a preferentially localize to the nucleus of post-mitotic neurons, where they might be involved in the regulation of gene expression in the nuclei of post-mitotic neurons. Recently, miR-25 was reported to inhibit the expression of the sarco(endo)plasmic reticulum Ca2 ATPase (SERCA2) by binding to the 3′-UTR of SERCA2 mRNA in the cytoplasm of post-mitotic neurons (Earls et al., 2012). It is therefore likely that miR-25, and possibly other nuclear-enriched miRNAs, are also involved in post-transcriptional gene regulation in mature neurons. A future challenge will be to specifically manipulate the nuclear and cytoplasmic pools of miRNAs to elucidate compartment-specific functions.

Since we observed a positive correlation between developmentally down-regulated and nuclear-enriched miRNAs, it is tempting to speculate that developmental stage-specific changes in biogenesis and/or degradation of these miRNAs might contribute to an enrichment of specific miRNAs in the nucleus. In addition to miRNA degradation in the cytoplasm, it is conceivable that targeting (or confinement) of miRNAs to the nucleus may be a mechanism to “remove” miRNAs from the cytoplasm to avoid regulation of cytoplasmic mRNA targets. Since the subcellular compartment of miRNA degradation remains unknown (Ruegger and Grosshans, 2012), it is possible that nuclear localization could be used to target miRNAs for degradation. Accordingly, some of the exoribonucleases such as ribosomal RNA-processing protein 41 (RRP41), exoribonuclease 1 (ERI-1) and 5′ to 3′ exoribonuclease XRN2, which are involved in miRNA degradation in metazoans (Ruegger and Grosshans, 2012), were shown to shuttle between the nucleus and cytoplasm (Ansel et al., 2008; Schmid and Jensen, 2008; Nagarajan et al., 2013) and participate in nuclear functions, e.g., ribosomal RNA biogenesis. A possible nuclear degradation of miRNAs is further supported by the observation that transfected siRNAs and endogenous miRNAs are enriched in the nucleolus (Ohrt et al., 2006; Politz et al., 2009). In this respect, studying the stability and localization of mature miRNAs upon their specific delivery into the nucleus or cytoplasm might help to identify the cellular compartment(s) important for degradation of mature miRNAs. Interestingly, it was shown that the turn-over of miRNAs in neurons can be regulated in an activity-dependent manner (Krol et al., 2010). It would be therefore important to determine the role of the nucleus in the rapid turnover of miRNAs in response to activity.

Irrespective of miRNA turnover, specific miRNAs (such as miR-25 and miR-92a) might perform distinct functions depending on the cell type and/or the developmental (metabolic) stage of a cell. For example, in neural stem cells and glial cells, some miRNAs repress neuron-promoting (and anti-proliferation) genes, e.g., by targeting the respective mRNAs and preventing their “leaky” expression in the cytoplasm. In post-mitotic neurons, the same miRNAs might be imported to the nucleus, where they could be involved in transcriptional or post-transcriptional regulation of gene expression as has been shown for some miRNAs and siRNAs in cancer cell lines (Kim et al., 2008; Place et al., 2008; Allo et al., 2009; Tang et al., 2012). In the future, the analysis of miRNA nucleo-cytoplasmic expression during differentiation of neural stem cells to fully differentiated neurons will be required to determine the exact time-point when the cytoplasmic function of specific neuronal miRNAs is switched to a function in the nucleus.

One of the mechanisms that miRNAs could employ to regulate transcriptional gene expression in the nucleus is by introducing epigenetic modification marks to DNA (methylation) and histone (acetylation and methylation) proteins. To clarify whether nuclear-enriched miRNAs are directly involved in epigenetic control of gene expression, specific manipulation of nuclear miRNAs followed by transcriptional and/or epigenetic profiling will be needed.

We also investigated whether cis-acting elements in mature miRNAs might direct them into the nucleus. Surprisingly, we identified that isomiRs, and to a smaller extent canonical miRNAs, containing 3′-terminal guanine nt are preferentially localized within the nucleus. In addition, we found that the source of the 3′-terminal G strongly influences nuclear fate. For example, isomiRs with NTA_G are the most nuclear enriched, followed by isomiR_Gs obtained from one 3′ nucleotide trimming, and then canonical isomiR_G for which the 3′-terminal is generated by Dicer/Drosha. IsomiR_Gs, independent of the source of the 3′-terminal guanine, could favor nuclear localization in at least two ways. First, the 3′-terminal guanine could confer higher stability in the nucleus. Second, binding of specific proteins to isomiR_Gs could mediate active transport from the cytoplasm to the nucleus. The active import of isomiR_Gs (as well as other isomiRs) to the nucleus might be performed by Argonaute proteins, which were shown to shuttle between the nuclear and cytoplasmic compartments (Weinmann et al., 2009; Nishi et al., 2013). Since Argonaute proteins (AGO 1–3) show different global small RNA binding pattern (Dueck et al., 2012), one could speculate that one of the AGO isoforms might specifically associate with isomiR_Gs and import them to the nucleus. In addition, RNA-binding proteins other than AGO might also be involved in nucleo-cytoplasmic shuttling of isomiRs. Non-templated addition of guanine to the 3′-terminus appears to further enhance nuclear accumulation. NTA_G could either happen in the nucleus after import, or in the cytoplasm followed by nuclear import. The identity of the guanylyltransferase(s) responsible for the production of NTA_Gs is unknown. Known metazoan RNA guanylyltransferases which are part of the mRNA cap-synthesis complex are unlikely to be involved in isomiR_G production, since these guanylyltransferases transfer a guanine monophosphate nucleoside to the nascent 5′ diphosphate mRNA end (Ghosh and Lima, 2010), but not the 3′ end. Apart from guanylation at the 3′ of miRNAs, the only example where terminal guanylyltransferase activity was observed is specific guanylation of European yellow lupine (*Lupinus luteus*) 5 s rRNA at the 3′ end in Hela cell extract (Wyszko et al., 1996). However, the responsible enzyme as well as physiological significance of this modification is not known. Interestingly, isomiRs with non-templated guanine addition are more abundant in mouse hippocampus (Zhou et al., 2012) and cerebellum (Wyman et al., 2011) compared to other tissues, suggesting that 3′ non-templated addition of guanine could be a brain-specific phenomenon. In this regard, determination of the identity and subcellular localization of guanylyltransferase responsible for NTA_G in neurons will be highly informative.

Taken together, our results indicate that mammalian neurons have a distinct subset of nuclear-enriched miRNAs, and that their localization to the nucleus might be linked to the developmental stage-specific down-regulation of miRNA expression. Furthermore, we uncovered that the type of nucleotide at the 3′-terminus of miRNA/isomiR can significantly influence subcellular localization of miRNAs in neurons. In the future, it will be important to characterize the physiological role of nuclear-enriched miRNAs in neurons, as well as the molecular mechanisms underlying nucleo-cytoplasmic localization, with a focus on the role of 3′-terminal guanylation. This will not only increase our understanding of neuronal development, but also provide important new insights into general aspects of miRNA metabolism.

## Author contributions

Sharof A. Khudayberdiev, Federico Zampa, and Marek Rajman performed experiments; Sharof A. Khudayberdiev performed data analysis (if not otherwise stated); Sharof A. Khudayberdiev and Gerhard Schratt wrote the manuscript; Gerhard Schratt supervised the project.

### Conflict of interest statement

The authors declare that the research was conducted in the absence of any commercial or financial relationships that could be construed as a potential conflict of interest.

## Supplementary material

The Supplementary Meterial for this article can be found online at: http://www.frontiersin.org/journal/10.3389/fnmol.2013.00043/abstract

Figure S1 **(A)** Cycle threshold (Ct) values for markers measured with qRT-PCR in Figure 1A. **(B)** Denaturing 15% PAGE gel showing equal loading of RNAs before membrane transfer for Northern blotting. The gel was stained with 2x SYBR Gold dye (Life Technologies) for 5 min and was imaged using E-BOX VX2 gel documentation system (PeqLab). **(C)** Northern blot analysis of miR-25 and miR-92a using cytoplasmic and nuclear RNA from neurons treated with KCl and BDNF. **(D)** MicroRNA ranking (Rank Sum) and distribution of an average developmental expression score (DES). DES was calculated by log_2_ transforming the ratio of miRNA read counts from prefrontal cortex of post-natal Day 3 (P3) and embryonic Day 10 (E10) rats in the published report of Yao et al. (2012). DES of 179 (out of 220) miRNAs that were detected both by us and Yao et al. (2012) were employed for analysis. The average DES (y-axis) was calculated using moving window technique, where window length was set as 10 and the average values were calculated by moving the window with one step at a time from high to low ranking miRNAs. In the x-axis, the ranking number of miRNAs in descending order is depicted.

Figure S2 **(A)** Immunostaining of primary hippocampal cultures for neuronal (MAP2, red) and astrocytic (GFAP, green) marker proteins after treatment with FUDR. Cultures were treated with 10 μM FUDR at DIV3 and fixed at DIV18. **(B)** Expression of indicated RNAs in mixed cultures and glia-enriched (10 % FBS-treated). The relative expression levels of indicated RNAs were obtained by the ddCt method. RNA levels in mixed cultures were arbitrarily set to 1. Bar plots show mean ± *SD* (*n* = 2). SD for mixed culture condition was determined after normalization to an internal control RNA (U6 snRNA). Statistical significance was determined based on U6 snRNA normalized values using Student's t-test with Bonferroni correction (^*^*p* < 0.05; ^**^*p* < 0.01).

Figure S3 **Sequence similarity of miRNAs**. Members of miRNA families possessing the same seed sequence are depicted in separate rectangular boxes. Red colored letters indicate conserved nucleotides between depicted family members. Blue colored letters indicate partial conservation (miR-25/92a/92b).

Figure S4 **Frequency of guanine (G, blue) and uracil (U, violet) nucleotides at the 3′-terminus of mature miRNAs (miRBase v18) depending on the NEnS**. Frequency of nucleotide was calculated using moving window technique, where window length was set as 20 and the average occurrence values were calculated by moving the window with one step at a time from high to low ranking miRNAs. In the x-axis, the ranking number of miRNAs in descending order is depicted.

Figure S5 **Proportion of specific isomiRs from the total sequence reads mapped to the respective miRNAs in the nuclear and the cytoplasmic fractions**.

Figure S6 **Relative NEnS and 3′-terminal guanine nucleotide of specific miRNA (miR-124 and miR-25) isomiRs**. On the y-axis, the log_2_ transformed read counts from nuclear fraction, and on the x-axis, rNEnS for each isomiR is depicted. Black colored bars indicate isomiRs that possess guanine nucleotide at the 3′-terminus.

Figure S7 **Impact of trimming on nuclear localization of isomiRs. (A)** rNEnS for canonical isomiR_A that underwent 3′ trimming, thereby exposing the indicated nucleotide. Bar plots show mean ± *SD* (canon_A, *n* = 75; trim_A>G, *n* = 25; trim_A>C, *n* = 18; trim_A>A, *n* = 8; trim_A>U, *n* = 10). Statistical significance was determined using Welch's *t*-test (unequal variance) with Bonferroni correction (^*^*p* < 0.05; ^**^*p* < 0.001; ^***^*p* < 1.0–E5). **(B)** rNEnS for canonical isomiR_C that underwent 3′ trimming, thereby exposing the indicated nucleotide. Bar plots show mean ± *SD* (canon_C, *n* = 62; trim_C>G, *n* = 13; trim_C>C, *n* = 17; trim_C>A, *n* = 2; trim_C>U, *n* = 9). Statistical significance was determined using Welch's *t*-test (unequal variance) and none of the comparisons passed the Bonferroni correction (^*^, *p* < 0.05).
