# Supplementary figures and images for: A comprehensive characterization of the nuclear microRNA repertoire of post-mitotic neurons
Source: Front Mol Neurosci. 2013 Nov 26;6:43. doi: 10.3389/fnmol.2013.00043 (PMC3840315; doi:10.3389/fnmol.2013.00043)

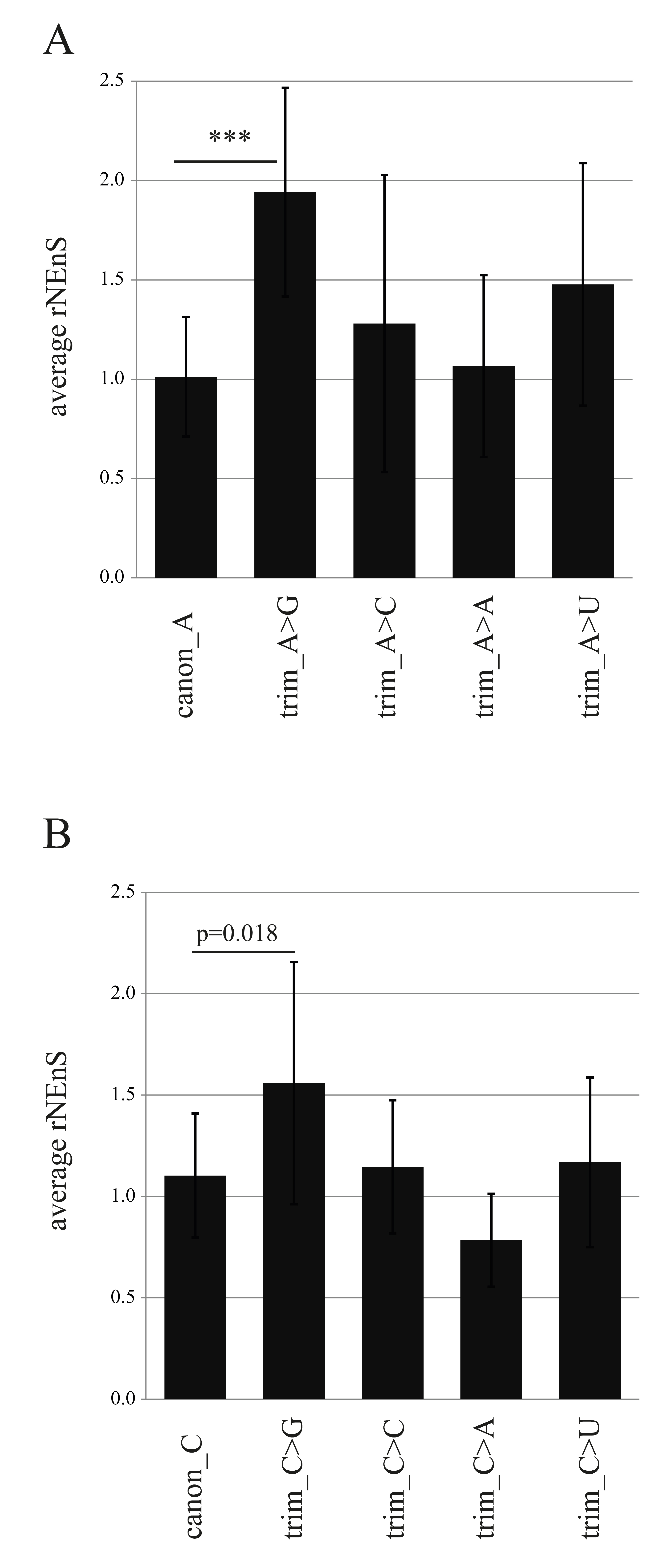

Supplement: Figure S1 — (A) Cycle threshold (Ct) values for markers measured with qRT-PCR in Figure 1A. (B) Denaturing 15% PAGE gel showing equal loading of RNAs before membrane transfer for Northern blotting. The gel was stained with 2x SYBR Gold dye (Life Technologies) for 5 min and was imaged using E-BOX VX2 gel documentation system (PeqLab). (C) Northern blot analysis of miR-25 and miR-92a using cytoplasmic and nuclear RNA from neurons treated with KCl and BDNF. (D) MicroRNA ranking (Rank Sum) and distribution of an average developmental expression score (DES). DES was calculated by log2 transforming the ratio of miRNA read counts from prefrontal cortex of post-natal Day 3 (P3) and embryonic Day 10 (E10) rats in the published report of Yao et al. (2012). DES of 179 (out of 220) miRNAs that were detected both by us and Yao et al. (2012) were employed for analysis. The average DES (y-axis) was calculated using moving window technique, where window length was set as 10 and the average values were calculated by moving the window with one step at a time from high to low ranking miRNAs. In the x-axis, the ranking number of miRNAs in descending order is depicted. [file Presentation1.ZIP › 68827_Schratt_Figure_15.JPEG]

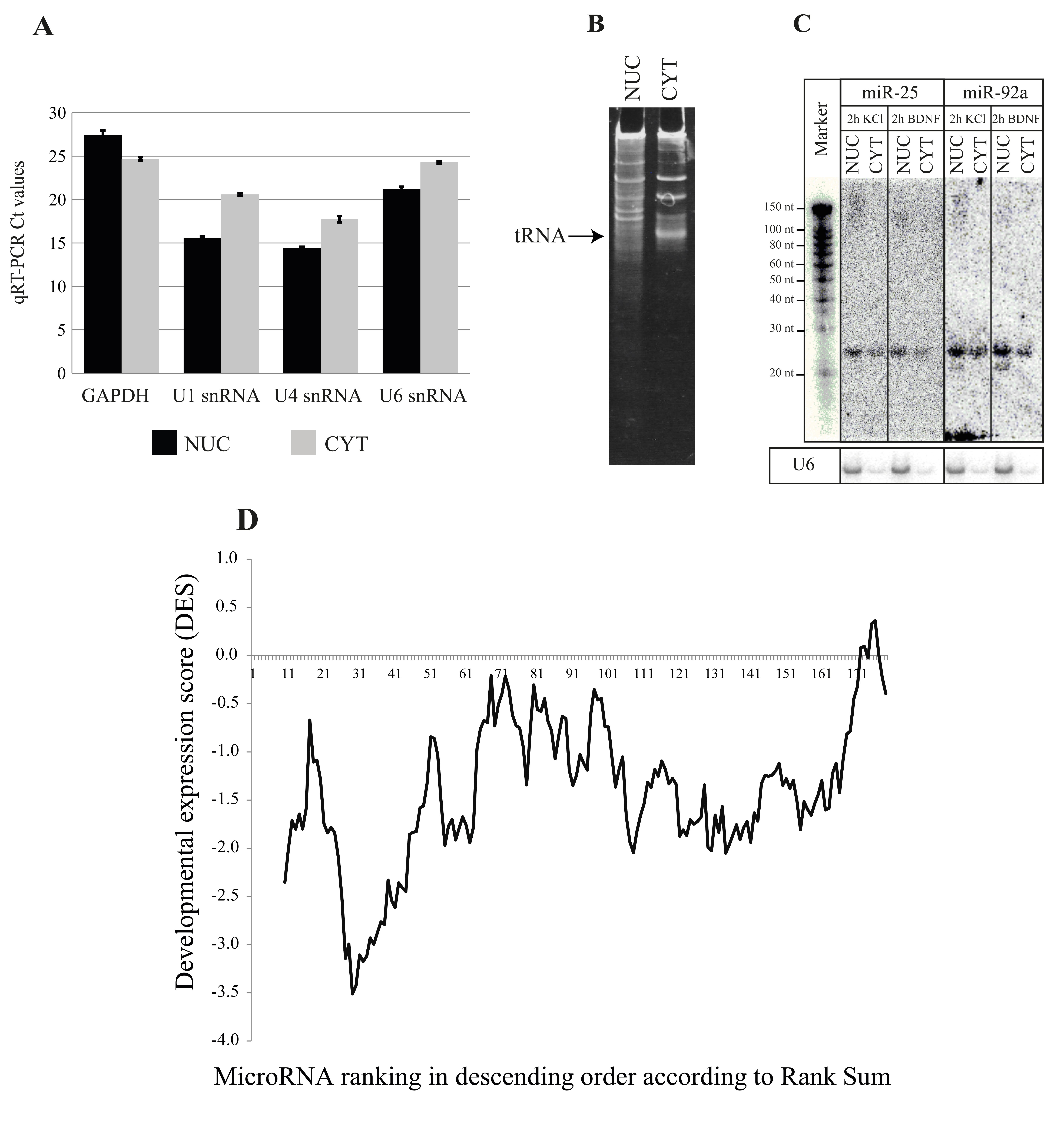

Supplement: Figure S1 — (A) Cycle threshold (Ct) values for markers measured with qRT-PCR in Figure 1A. (B) Denaturing 15% PAGE gel showing equal loading of RNAs before membrane transfer for Northern blotting. The gel was stained with 2x SYBR Gold dye (Life Technologies) for 5 min and was imaged using E-BOX VX2 gel documentation system (PeqLab). (C) Northern blot analysis of miR-25 and miR-92a using cytoplasmic and nuclear RNA from neurons treated with KCl and BDNF. (D) MicroRNA ranking (Rank Sum) and distribution of an average developmental expression score (DES). DES was calculated by log2 transforming the ratio of miRNA read counts from prefrontal cortex of post-natal Day 3 (P3) and embryonic Day 10 (E10) rats in the published report of Yao et al. (2012). DES of 179 (out of 220) miRNAs that were detected both by us and Yao et al. (2012) were employed for analysis. The average DES (y-axis) was calculated using moving window technique, where window length was set as 10 and the average values were calculated by moving the window with one step at a time from high to low ranking miRNAs. In the x-axis, the ranking number of miRNAs in descending order is depicted. [file Presentation1.ZIP › 68827_Schratt_Figure_9.JPEG]

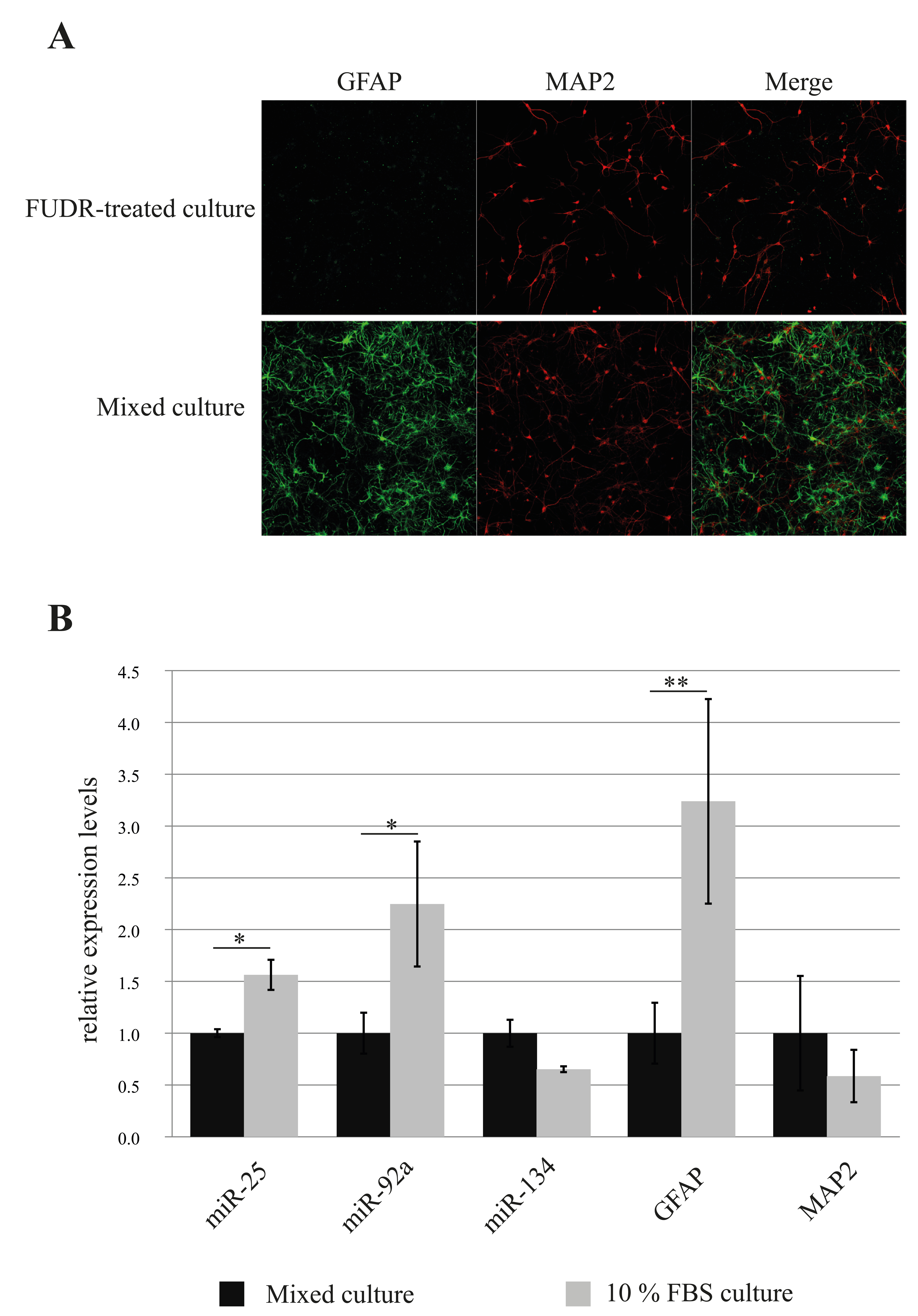

Supplement: Figure S1 — (A) Cycle threshold (Ct) values for markers measured with qRT-PCR in Figure 1A. (B) Denaturing 15% PAGE gel showing equal loading of RNAs before membrane transfer for Northern blotting. The gel was stained with 2x SYBR Gold dye (Life Technologies) for 5 min and was imaged using E-BOX VX2 gel documentation system (PeqLab). (C) Northern blot analysis of miR-25 and miR-92a using cytoplasmic and nuclear RNA from neurons treated with KCl and BDNF. (D) MicroRNA ranking (Rank Sum) and distribution of an average developmental expression score (DES). DES was calculated by log2 transforming the ratio of miRNA read counts from prefrontal cortex of post-natal Day 3 (P3) and embryonic Day 10 (E10) rats in the published report of Yao et al. (2012). DES of 179 (out of 220) miRNAs that were detected both by us and Yao et al. (2012) were employed for analysis. The average DES (y-axis) was calculated using moving window technique, where window length was set as 10 and the average values were calculated by moving the window with one step at a time from high to low ranking miRNAs. In the x-axis, the ranking number of miRNAs in descending order is depicted. [file Presentation1.ZIP › 68827_Schratt_Figure_10.JPEG]

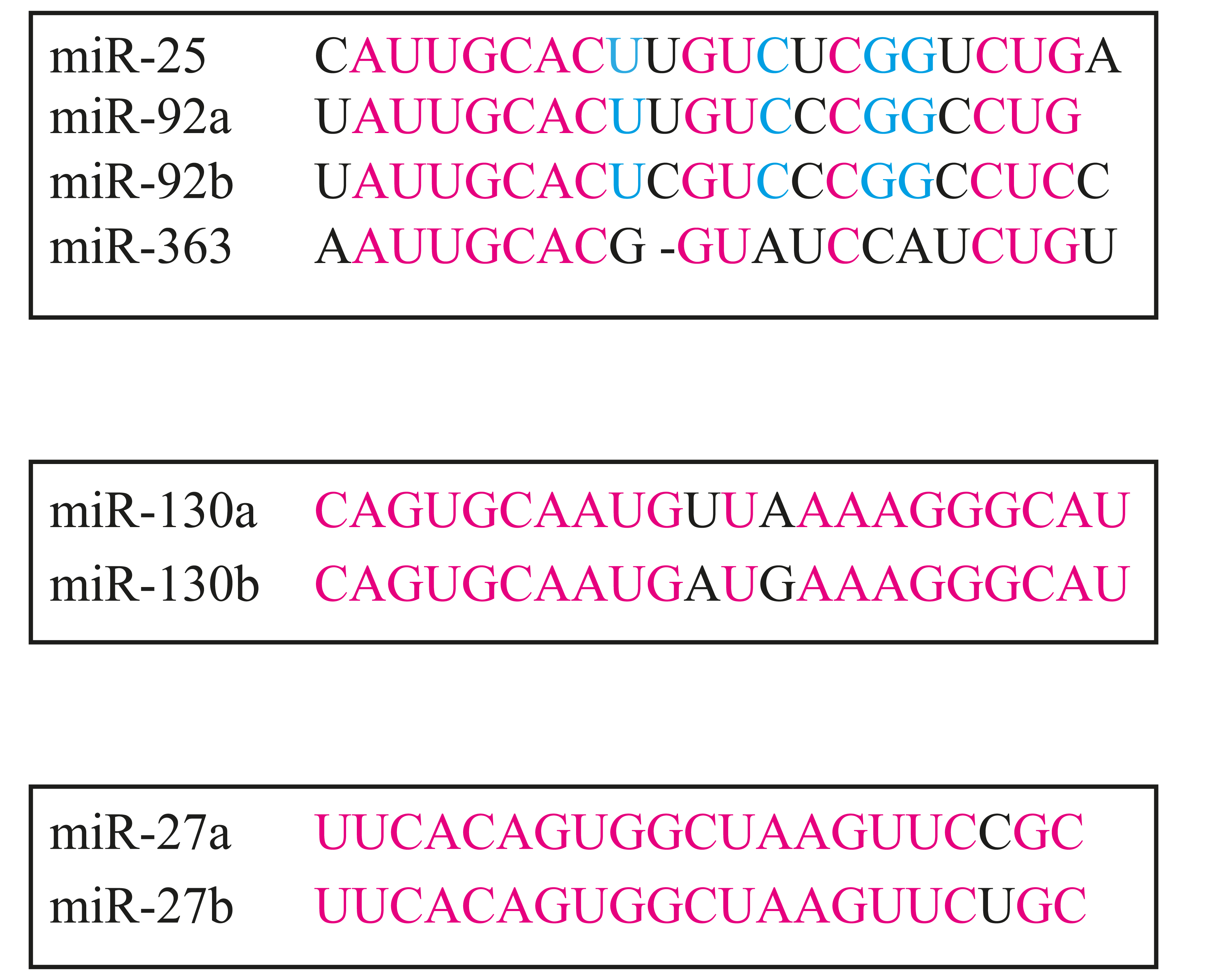

Supplement: Figure S1 — (A) Cycle threshold (Ct) values for markers measured with qRT-PCR in Figure 1A. (B) Denaturing 15% PAGE gel showing equal loading of RNAs before membrane transfer for Northern blotting. The gel was stained with 2x SYBR Gold dye (Life Technologies) for 5 min and was imaged using E-BOX VX2 gel documentation system (PeqLab). (C) Northern blot analysis of miR-25 and miR-92a using cytoplasmic and nuclear RNA from neurons treated with KCl and BDNF. (D) MicroRNA ranking (Rank Sum) and distribution of an average developmental expression score (DES). DES was calculated by log2 transforming the ratio of miRNA read counts from prefrontal cortex of post-natal Day 3 (P3) and embryonic Day 10 (E10) rats in the published report of Yao et al. (2012). DES of 179 (out of 220) miRNAs that were detected both by us and Yao et al. (2012) were employed for analysis. The average DES (y-axis) was calculated using moving window technique, where window length was set as 10 and the average values were calculated by moving the window with one step at a time from high to low ranking miRNAs. In the x-axis, the ranking number of miRNAs in descending order is depicted. [file Presentation1.ZIP › 68827_Schratt_Figure_11.JPEG]

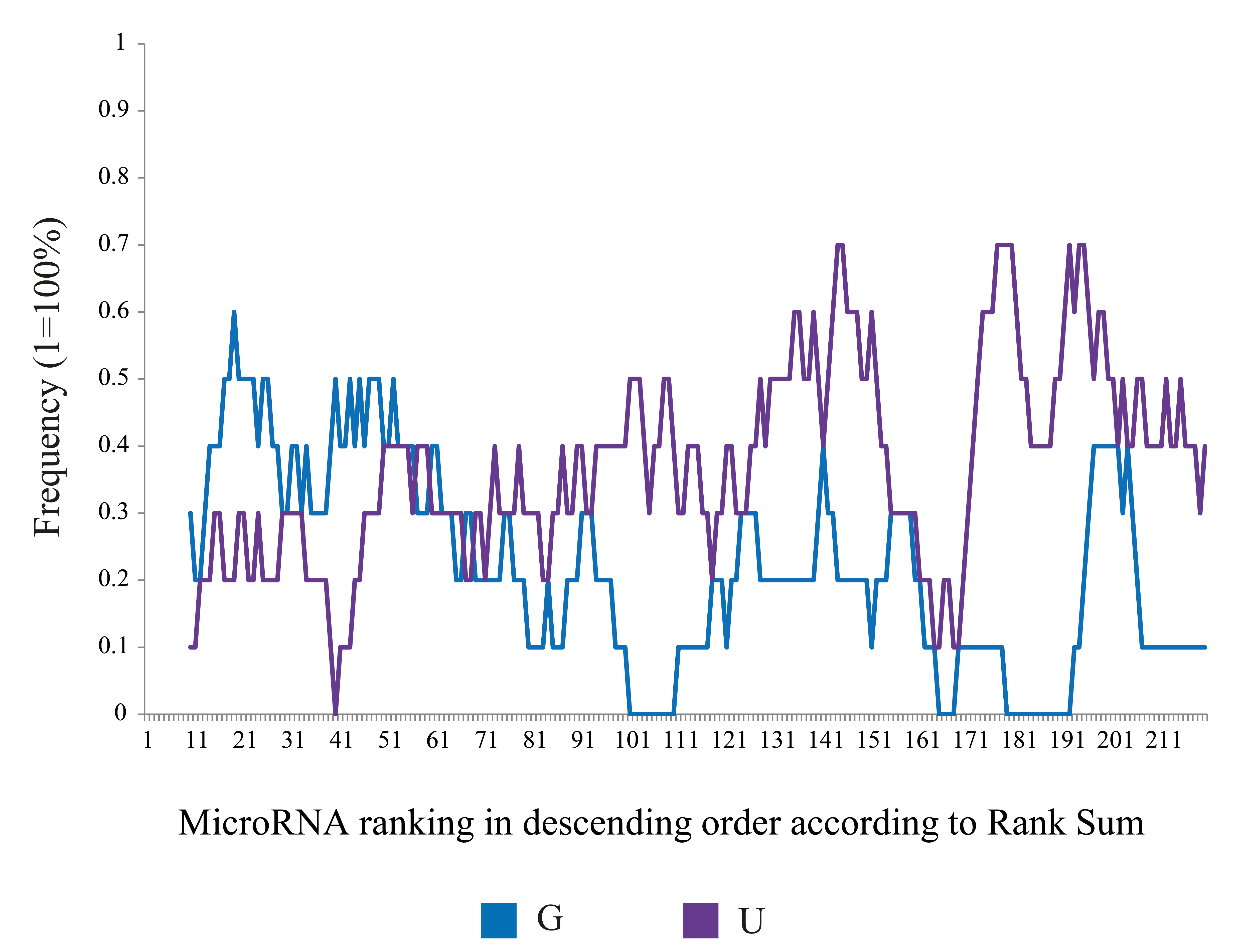

Supplement: Figure S1 — (A) Cycle threshold (Ct) values for markers measured with qRT-PCR in Figure 1A. (B) Denaturing 15% PAGE gel showing equal loading of RNAs before membrane transfer for Northern blotting. The gel was stained with 2x SYBR Gold dye (Life Technologies) for 5 min and was imaged using E-BOX VX2 gel documentation system (PeqLab). (C) Northern blot analysis of miR-25 and miR-92a using cytoplasmic and nuclear RNA from neurons treated with KCl and BDNF. (D) MicroRNA ranking (Rank Sum) and distribution of an average developmental expression score (DES). DES was calculated by log2 transforming the ratio of miRNA read counts from prefrontal cortex of post-natal Day 3 (P3) and embryonic Day 10 (E10) rats in the published report of Yao et al. (2012). DES of 179 (out of 220) miRNAs that were detected both by us and Yao et al. (2012) were employed for analysis. The average DES (y-axis) was calculated using moving window technique, where window length was set as 10 and the average values were calculated by moving the window with one step at a time from high to low ranking miRNAs. In the x-axis, the ranking number of miRNAs in descending order is depicted. [file Presentation1.ZIP › 68827_Schratt_Figure_12.JPEG]

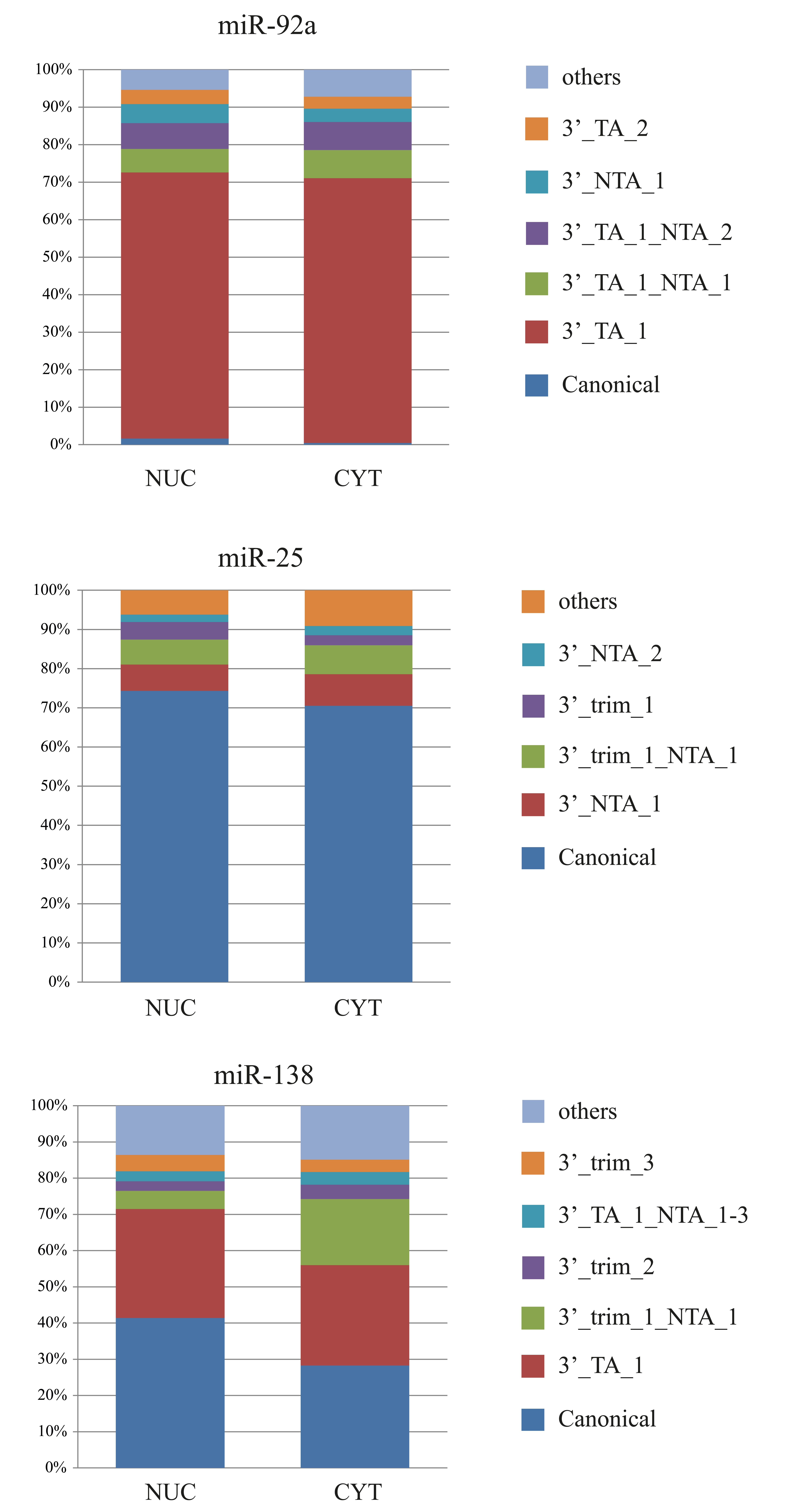

Supplement: Figure S1 — (A) Cycle threshold (Ct) values for markers measured with qRT-PCR in Figure 1A. (B) Denaturing 15% PAGE gel showing equal loading of RNAs before membrane transfer for Northern blotting. The gel was stained with 2x SYBR Gold dye (Life Technologies) for 5 min and was imaged using E-BOX VX2 gel documentation system (PeqLab). (C) Northern blot analysis of miR-25 and miR-92a using cytoplasmic and nuclear RNA from neurons treated with KCl and BDNF. (D) MicroRNA ranking (Rank Sum) and distribution of an average developmental expression score (DES). DES was calculated by log2 transforming the ratio of miRNA read counts from prefrontal cortex of post-natal Day 3 (P3) and embryonic Day 10 (E10) rats in the published report of Yao et al. (2012). DES of 179 (out of 220) miRNAs that were detected both by us and Yao et al. (2012) were employed for analysis. The average DES (y-axis) was calculated using moving window technique, where window length was set as 10 and the average values were calculated by moving the window with one step at a time from high to low ranking miRNAs. In the x-axis, the ranking number of miRNAs in descending order is depicted. [file Presentation1.ZIP › 68827_Schratt_Figure_13.JPEG]

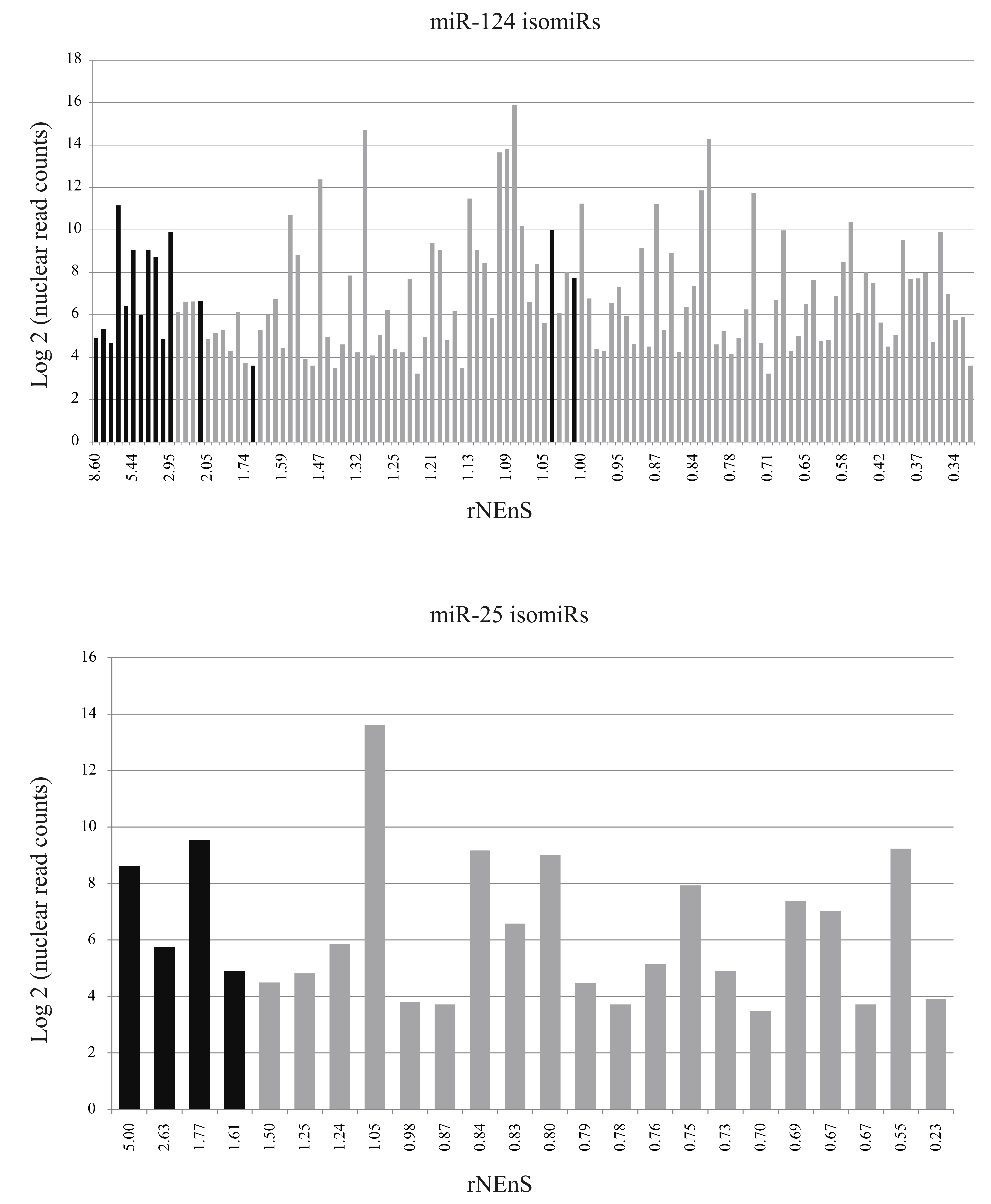

Supplement: Figure S1 — (A) Cycle threshold (Ct) values for markers measured with qRT-PCR in Figure 1A. (B) Denaturing 15% PAGE gel showing equal loading of RNAs before membrane transfer for Northern blotting. The gel was stained with 2x SYBR Gold dye (Life Technologies) for 5 min and was imaged using E-BOX VX2 gel documentation system (PeqLab). (C) Northern blot analysis of miR-25 and miR-92a using cytoplasmic and nuclear RNA from neurons treated with KCl and BDNF. (D) MicroRNA ranking (Rank Sum) and distribution of an average developmental expression score (DES). DES was calculated by log2 transforming the ratio of miRNA read counts from prefrontal cortex of post-natal Day 3 (P3) and embryonic Day 10 (E10) rats in the published report of Yao et al. (2012). DES of 179 (out of 220) miRNAs that were detected both by us and Yao et al. (2012) were employed for analysis. The average DES (y-axis) was calculated using moving window technique, where window length was set as 10 and the average values were calculated by moving the window with one step at a time from high to low ranking miRNAs. In the x-axis, the ranking number of miRNAs in descending order is depicted. [file Presentation1.ZIP › 68827_Schratt_Figure_14.JPEG]
